# Thinking climate change through the lens of abstractness: a multi-task and multi-setting investigation into generational differences in the conceptualization of ecology

**DOI:** 10.1186/s41235-025-00689-4

**Published:** 2025-11-16

**Authors:** Ilenia Falcinelli, Chiara Fini, Claudia Mazzuca, Guido Alessandri, Fabio Alivernini, Roberto Baiocco, Andrea Chirico, Lorenzo Filosa, Tommaso Palombi, Jessica Pistella, Simone Tavolucci, Fabio Lucidi, Anna M. Borghi

**Affiliations:** 1 Department of Dynamic and Clinical Psychology, and Health Studies, Faculty of Medicine and Psychology, Sapienza University of Rome, Rome, Italy; 2 Department of Psychology, Faculty of Medicine and Psychology, Sapienza University of Rome, Rome, Italy; 3 Department of Developmental and Social Psychology, Faculty of Medicine and Psychology, Sapienza University of Rome, Rome, Italy; 4 Institute of Cognitive Sciences and Technologies, Italian National Research Council, Rome, Italy

**Keywords:** Ecology, Abstractness, Conceptualization, Older adults, Younger adults, Exposure to nature

## Abstract

To face ecological disasters, one of the major emergencies of modern times, fostering pro-environmental behaviors appears crucial. While research explored various behavioral drivers, few studies addressed the conceptual representation of ecological events at different ages. Our preregistered study fills this gap, examining how different generations conceptualize ecology, considering evidence suggesting a link between climate change abstractness and willingness to act pro-environmentally. Older (> 65 yo) and younger (18–35 yo) individuals performed multiple tasks targeting conceptual processing (categorization), semantic organization (rating), and conceptual representation (feature generation) of ecological (e.g., *deforestation*) compared to concrete (e.g., *spoon*) and abstract (e.g., *affirmation*) concepts. In the categorization task, participants responded to critical ecological, concrete, and abstract words but refrained from responding to animal words. The rating task involved evaluating critical words on semantic aspects (e.g., Abstractness, Familiarity), and the feature generation task listing properties true for each concept. Innovatively, participants were tested in three possible settings (indoor, natural outdoor, urbanized outdoor) to test whether exposure to nature impacts categorization.

Results showed that independent from the setting, ecological concepts were similar to abstract concepts, especially in semantic organization and conceptual representation, or more abstractly characterized than them, especially in conceptual processing. Age differences were minimal, but older adults, who showed more positive attitudes toward ecology and nature, characterized ecological concepts more concretely than younger adults.

Theoretically, the multifarious characterization of ecological phenomena supports the flexibility of our conceptual system and points to the necessity of overcoming the classical *concrete-abstract* dichotomy and studying concepts in a more “situated” manner. From a societal point of view, the age-dependent abstractness of ecological concepts might inform policies on how to improve climate change campaigns, tailoring them to different age groups.

**Supplementary Information:**

The online version contains supplementary material available at 10.1186/s41235-025-00689-4.

## Introduction

The ecological emergency has become a crucial object of debate in contemporary Western societies. Numerous studies in biology, engineering, demography, and other disciplines focused on the causes and consequences of this phenomenon. However, a pivotal key to reducing damage to the environment is human behavior (Ferguson & Schmitt, 2021; Sinclair et al., 2025). In psychology, some studies addressed individual factors—beliefs, motivations, and subjective norms—leading to pro-environmental behaviors, such as recycling (review in Li et al., 2019). Other studies focused on risk perception and affective responses to ecological disasters and on collective behaviors and norms (review in Tam et al., 2021).

The literature on concepts, i.e., on how people think and what they know about the domain related to ecological issues, instead, is extremely scarce (for an exception, Malt & Majid, 2023). C*oncepts* are the minimal units of knowledge, which link past, present, and future experiences, allow classifying entities and objects and drawing inferences (Kemmerer, 2023; Murphy, 2002). The most common way to investigate concepts is studying the meaning of the words expressing them (Barsalou, 1999). Recent studies focus on whether people feel part of the nature (Pizza & Deborah, 2023; Flusberg & Thibodeau, 2023), how people from different cultures conceptualize natural elements, like landscapes (Striedl et al., 2024; van Putten et al., 2020), or forests (Burenhult et al., 2017) (review in Falcinelli et al., 2024c), or test what people know about ecological issues (Ranney & Clark, 2016). However, to our knowledge, no study directly focuses on concepts we have called “ecological”, such as *ozone hole*,^1^*climate change* and *pollution*, which refer to the ecological emergency (for an exception, Falcinelli et al., 2024a).

Interestingly, a branch within the psychological field has addressed the relationship between ecological events and age groups. Some contributions investigated the effects of ecological issues on well-being (Tsevreni et al., 2023), focusing on eco-anxiety, a new form of distress caused by the unpredictability and uncontrollability of ecological disasters (Pihkala, 2020). Younger adults show higher levels of eco-anxiety than older adults and express greater concerns about ecological issues (APA, 2018; Hill-Harding et al., 2025). Conversely, ecological phenomena generate more physical consequences for older adults since it can exacerbate pre-existing medical conditions (e.g., air pollution can aggravate breathing problems—Ayalon et al., 2023).

Also, the studies on the relationships between intentions, motivations, and pro-environmental behaviors have highlighted intergenerational differences. Younger adults are more interested in ecological topics (Clayton, 2020), have more pro-environmental attitudes (Wiernik et al., 2013), and are more willing to engage in climate activism than older adults (Ballew et al., 2020). Daily, older people more frequently engage in pro-environmental behaviors (Wang et al., 2021), such as using less electric power, recycling more, and purchasing more carefully (López-Mosquera et al., 2015) and also show more positive habits toward nature than younger adults (Scott et al., 2015). Overall, younger people’s “call to action” expresses more ideologically (activism) than in terms of actual eco-friendly behaviors (Ágoston et al., 2024)*.* This difference might occur because younger adults may feel more helpless and perceive environmental action as primarily the responsibility of powerful entities like politicians (Sarrasin et al., 2022). Conversely, older adults, transitioning from full workers to retired, might increase their prosocial behaviors (review in Georganas et al., 2022).

Curiously, despite the divergences in the psycho-physical well-being, attitudes, and behaviors, a comprehensive review reports limited differences between younger and older people in the knowledge concerning climate change (Corner et al., 2015).

Innovatively, our study allows us to understand whether the different ways older and younger adults deal with ecological emergencies are due to different ways to conceive ecological concepts—for example, intending them as more abstract or concrete. Several contributions provide evidence on age-dependent variations in conceptualization, revealing significant differences not only in the quantity (Dubossarsky et al., 2017), structure (Cosgrove et al., 2021), and content of knowledge related to a conceptual domain (Vignando et al., 2018), but also in its level of abstractness (review in Borghi & Setti, 2017). In addition, a recent work shows that, regardless of age, the more people have experience and familiarity—two key points of concreteness (Barca et al., 2002; Mazzuca et al., 2025a, 2025b; Villani et al., 2022)—with “climate change”, the more coherent and consistent the mental models brought to cope with that problem are likely to be (Bostrom, 2017). Taken together, this evidence suggests that testing changes in the level of abstractness of ecological concepts across age groups may help deepen our understanding of the development of pro-environmental attitudes and behaviors across the lifespan and implement efficient age-tailored interventions to cope with climate change.

So, in this work, for the first time, we address how different generations conceive ecological concepts, and we did so by employing multiple tasks inspired by cognitive studies on categorization. Theoretically, aside from the possible impact of this study in everyday life, investigating ecological concepts can advance research on categorization for various aspects. First, because the extensive use of ecological concepts is relatively recent, they are ideal to understand whether concepts are flexible and to what extent the introduction of new concepts might lead to reorganizing people’s semantic networks. Similarly, the new concept of COVID-19 led people to rearrange their semantic representation of illness (Mazzuca et al., 2022).

Second, the members of ecological concepts cut across categorical boundaries between natural kinds (e.g., *tree*) and artifacts (e.g., *hammer*)—for example, climate, a natural phenomenon, changed due to human intervention. Thus, their study can contribute to a more fine-grained representation of the differences between concept kinds.

Third, ecological concepts are intriguing because they cut across the classic distinction between concrete concepts (e.g., *tree, hammer*), typically evoking more sensorimotor experiences, and abstract concepts (e.g., *freedom, thought*), for which linguistic, inner, and social experience play a major role (Borghi, 2023, 2025; Mazzuca et al., 2025a, 2025b). A recent rating study shows that ecological concepts figure as “hybrid”: For some aspects, they are similar to concrete concepts, while for others, people consider them as equally abstract or even more abstract than abstract concepts (Falcinelli et al., 2024a; see also Fini et al., 2025). Here, we test whether they differ from abstract and concrete concepts in their semantic organization, representation and processing, using ratings, feature generation, and categorization tasks. Testing their hybrid character may be critical since, according to some positions (e.g., Leviston et al., 2014), conceptualizing the ecological domain as abstract rather than concrete can explain the resistance to conduct direct pro-environmental actions.

Aside from research on categorization, our study is also motivated by research on the impact of nature on cognition (Cassarino & Setti, 2015). Different theories have underlined the restorative power of the contact with nature (Kaplan 1995), and the positive memories associated with it (Egner et al., 2000). According to the attention restoration theory (Kaplan, 1995; Schertz & Berman, 2019), immersion in a natural context favors attention focusing due to the reduced stimuli overload (Linnell et al., 2013). Being exposed also shortly to natural contexts favors sustained attention in younger adults, and older adults benefit from the exposure to nature for attention (Sia et al., 2020; Jarosz, 2023; but see Cassarino et al., 2019). The cognitive performances, especially of older adults, are better in natural than in indoor or urbanized outdoor settings (e.g., Hartig et al., 2003; Kaplan, 1995; Sia et al., 2020; Tan et al., 2019). There is, however, also contrasting evidence suggesting that urbanized environments, even if crowded with noisy stimuli, represent a source of multi-sensorial stimulation and brain training for older adults (Cassarino & Setti, 2015). While research has focused on attention, to our knowledge, no study has investigated the effects of exposure to an outdoor or indoor setting on categorization, as we do here. On a more theoretical perspective—in keeping with the embodied-enactive-embedded-extended view (Borghi, 2023; Groth & Nimkulrat, 2024) and the Material Engagement Theory (Malafouris, 2013)—concepts may vary depending on the environmental and social interactions in which individuals are here-and-now engaged (Barsalou et al., 2018). Assuming short-term or immediate contexts can modulate conceptual categorization implies acknowledging an even deeper level of semantic flexibility.

### Our study

In our study, we address how Italian older (over 65) and younger (18–35) individuals conceptualize the ecological domain, by targeting conceptual processing, semantic organization, and representation through a categorization, a rating and a feature generation task. Across tasks, experimental stimuli were ecological concepts (e.g., *pollution*) along with abstract (e.g., *definition*) and concrete concepts (e.g., *umbrella*), used as contrasting categories to explore the hypothesized *hybrid* nature of the former.

In the categorization task (Barca et al., 2020), participants read our concepts of interest, i.e., ecological, abstract, and concrete concepts (from now on, “critical concepts”), along with animal concepts, and were required to refrain from responding to animal concepts (or astrological concepts—Online Appendix A, Supplementary Materials, from now on, “SM”). We examined reaction times (RTs) of older and younger adults for ecological compared to abstract and concrete concepts, considering evidence documenting a processing advantage of concrete over abstract concepts (Concreteness Effect—Paivio, 1990).

In the rating task (Brysbaert et al., 2014; Lynott et al., 2020), we investigated conceptual organization by asking participants to evaluate ecological, abstract, and concrete concepts on dimensions targeting relevant aspects to define abstractness using Likert scales, and then, we explored whether ecological concepts appeared more similarly characterized to abstract or concrete concepts across age groups.

Finally, the feature generation task (Borghi & Barsalou, 2021; Santos et al., 2011) required participants to list all features they considered true for ecological, abstract, and concrete concepts. We investigated conceptual representations analyzing the number of listed features for the three conceptual kinds. In addition, to enrich the previous literature on age-dependent differences in the content of ecological knowledge (Corner et al., 2015—Sect. “Introduction”), we also deepened our investigation by exploring the content of features generated by older and younger adults for ecological concepts, by focusing on the most representative concept within our wordpool.

Innovatively, we tested both younger and older participants across three distinct environmental settings: indoor (a laboratory or a room within a house), naturalistic outdoor (a park or garden), and urban outdoor (a public square or a house’s balcony). This design aimed to investigate whether variations in environmental affordances and multisensory stimulation influence semantic processing across age groups (Sects. “Introduction” and “Procedure”).

In line with practices of Open Science, we preregistered the study at the beginning of data collection (except for the “control” categorization task— Online Appendix A, SM), and we accessed data just once it ended. The Preregistered Plan can be accessed at this OSF registry link: 10.17605/OSF.IO/CV3E2.

### Experimental hypotheses^2^

#### Conceptual processing (Categorization task)

##### H1

Across types of concepts and settings, older adults should show slower RTs than younger adults due to the cognitive decline related to aging (Hultsch et al., 2002).

##### H2

We anticipated three possible scenarios characterizing ecological compared to abstract and concrete concepts in RTs:

##### H2.1

If they are at the border between abstract and concrete concepts, they should be in the middle between abstract (slowest RTs) and concrete concepts (fastest RTs—see Concreteness Effect, Sect. “Our study”).

##### H2.2

If they are characterized similarly to abstract concepts, they should be as fast as abstract concepts and slower than concrete concepts in RTs.

##### H2.3

If they are characterized more abstractly than abstract concepts, they should be slower than abstract (and concrete) concepts in RTs.

##### H3

Considering literature on contextual effect on cognitive performances (Sect. “Introduction”), we expected differences in RTs between the three settings. If the exposition to nature impacts categorization, the natural environment should have an advantage over the other conditions. If the simple fact of being outdoors enhances performance, we expected an RTs advantage in the two outdoor conditions over the indoor one. Instead, if less noisy environments facilitate performances, the urbanized outdoor condition should be disadvantaged compared to the other conditions.

##### H4

Based on previous evidence (Sect. “Introduction”), we hypothesized older adults would benefit more—in terms of RTs—than younger adults from the natural outdoor (and perhaps the urbanized outdoor) compared to the indoor condition and more from the natural outdoor compared to the urbanized condition.

#### Conceptual organization (rating task)

##### H5

We anticipated three possible semantic patterns characterizing ecological compared to abstract and concrete concepts:

##### H5.1

They could lie for most of the semantic dimensions in the middle between abstract and concrete concepts.

##### H5.2

They could be more similar to abstract concepts, i.e., their scores on most dimensions could differ from concrete concepts but not from abstract concepts.

##### H5.3

They could display a more abstract characterization than abstract (and concrete) concepts, i.e., their scores on most dimensions could be more abstract than those to abstract and concrete concepts.

##### H6

Younger adults, due to their higher pro-environmental attitudes, should characterize ecological concepts less abstractly than older adults.

##### H7

Based on H6 hypothesis, younger adults should more frequently characterize ecological at the border between abstract and concrete concepts (H5.1) or similarly to abstract concepts (H5.2); conversely, older adults should conceive them as similar to abstract concepts (H5.2) or more abstract than abstract concepts (H5.3).

#### Conceptual representation (Feature generation task)

##### H8

Based on previous literature on age-dependent differences in eco-attitudes (H6 hypothesis and Sect. “Introduction”), younger adults should have a richer representation of the ecological domain than older adults. Thus, by looking at their production for the most representative concept:

##### H8.1

They should generate more complex, diversified, and experience-driven features than older adults.

##### H8.2

More broadly, the knowledge content associated with the concept should differ between the two age groups.

##### H9

In line with literature suggesting that abstract concepts, having a less dense representation in semantic memory, make related knowledge harder to access than concrete concepts (Recchia & Jones, 2012; Yap & Pexman, 2016), we expected both age groups to list fewer properties for abstract than concrete concepts. In addition, because abstract concepts’ meaning varies more across individuals (Borghi & Mazzuca, 2023; Wang & Bi, 2021), there should be more unique features (i.e., properties generated just by one participant), for abstract than concrete concepts (Canessa et al., 2021).

For ecological concepts, we anticipated three possible scenarios:

##### H9.1

If they lie in the middle between abstract and concrete concepts, participants should produce more properties and less unique features than for abstract concepts and fewer properties and more unique features than for concrete concepts.

##### H9.2

If they are more similar to abstract concepts, participants should produce fewer properties and more unique features for ecological than concrete concepts and both in a similar number as for abstract concepts.

##### H9.3

If they display a more abstract pattern than abstract concepts, participants should produce fewer properties and more unique features for ecological than for both abstract and concrete concepts.

#### Differences across age cohorts in their attitudes toward ecology and nature.

##### H10

Based on field literature (Sect. “Introduction”), we expected older and younger adults to differ *a-priori* in their attitudes toward ecology and nature.

#### Data availability

Experimental materials, raw data, and analysis scripts are available at the following OSF repository: 10.17605/OSF.IO/V8XC9.

## Methods

### Participants

To estimate the sample size for the study, we used MorePower (version 6.0.4, Campbell & Thompson, 2012). Similar studies on older adults report large effect sizes (e.g., Vignando et al., 2018, η_p_^2^ = 0.34), but we reasoned that decreasing them could help us control for not considered variables potentially affecting the experiment (Larranaga & Sereno, 2023). So, we calculated the number of participants required to achieve a *medium* effect size (η_p_^2^ = 0.06), with a power of 80%, an alpha error of 0.05, and a correlation among repeated measures of .50 for an ANOVA with a within factor of three levels (Category of Word: Abstract, Concrete, Ecological), two between factors of two and three levels (Group: Older, Younger; Setting: Indoor, Natural Outdoor, Urbanized Outdoor), and their interaction. This resulted in 96 participants.

The final sample was therefore composed of 48 older (30 females, *M*_*age*_ = 72.38; *SD*_*age*_ = 6.16; *Range*_*age*_ = 65–87) and 48 younger adults (20 females, *M*_*age*_ = 23.94; *SD*_*age*_ = 2.32; *Range*_*age*_ = 19–28).

We recruited volunteer participants through word-of-mouth/snowballing. The inclusion criteria were being an Italian native speaker, aged between 18 and 35 for the youngest, and equal or over 65 for the older sample (Orimo et al., 2006). To ensure efficient cognitive functioning, older participants completed the Mini-Mental State Examination (MMSE—Folstein et al., 1975) before the experiment; none reported cognitive impairment (MMDE: *M* = 29.23; *SD* = 1.34). Table S1, SM reports socio-demographic information.

### Materials

#### Words

*Words selection.* The stimuli (*N* = 160) consisted of ecological, abstract, and concrete concepts (*n* = 40 each). Abstract concepts encompassed philosophical-spiritual concepts (e.g., *religion*), i.e., the most abstract words identified in the Italian literature on concepts (Villani et al., 2019), while concrete concepts contained artifacts (e.g., *statue*). In the categorization tasks, we also included 40 animal words as fillers (e.g., *monkey*) (Barca et al., 2020).

We selected the critical concepts from recent Italian databases: ecological concepts from Falcinelli et al.’s (2024a), abstract concepts from Villani et al.’s (2019), and concrete concepts from Della Rosa et al.’s (2010). Differently, we selected animal concepts through a consultation among ourselves.

Critical concepts were matched by frequency of use and word length. We manually calculated the length of critical words and retrieved word frequency scores from Google Ngram Viewer (Weiss, 2015). Two separate ANOVAs showed no differences across conceptual kinds in Word Length, *F*(2, 117) = 2.60, *p* = 0.079 (Abstract: *M* = 9.00, *SE* = 0.33; Concrete: *M* = 8.12, *SE* = 0.33; Ecological: *M* = 9.10, *SE* = 0.33) and Frequency of Use, *F*(2, 117) = 2.39, *p* = 0.096 (Abstract: *M* = 0.02%, *SE* = 0.01%; Concrete: *M* = 0.01%, *SE* = 0.01%; Ecological: *M* = 0.02%, *SE* = 0.01%).

*Stimuli.* The stimuli of the categorization task comprised 40 concepts for each kind (abstract, concrete, ecological, animal). The feature generation and rating tasks included 10 abstract, 10 concrete, 10 ecological concepts, and no animal concept. Abstract concepts were the 10 with the highest Concreteness ~ Abstractness scores (*M* = 5.32; *SD* = 0.42) in Villani et al.’s (2019) norms, and concrete concepts were the 10 with the lowest Concreteness ~ Abstractness scores (*M* = 1.04; *SD* = 0.02) in Della Rosa et al.’s (2010) norms. We selected as Ecological concepts those rated on a Likert scale (1 = “*very little*”; 7 = “*very much*”) as the 10 most representative of the ecological domain by 23 independent participants (*M* = 5.97; *SD* = 0.33) (for an overview of the stimuli, Tables S2 and S3, SM).

#### Semantic dimensions

In the rating task, participants evaluated ecological, abstract, and concrete concepts on seven semantic dimensions using 7-point Likert scales.

Some dimensions were the more frequently employed in literature on abstract concepts—i.e., Abstractness ~ Concreteness (Paivio, 1990); Age of Acquisition (Gilhooly & Logie, 1980), i.e., the estimated age at which participants have acquired a concept/word; Familiarity, indicating the perceived level of personal experience with a concept (Barca et al., 2020). Other dimensions targeted metacognitive aspects: Social Metacognition evaluated to what extent individuals feel the need to rely on others to understand conceptual meaning (Borghi, 2022) and Word Confidence indicated individuals’ certainty to master word meanings (Mazzuca et al., 2022). Further properties related to social/communicative characteristics: Openness to Negotiation indicated how negotiable participants feel conceptual meanings are (Fini et al., 2023), and Perceived Distance the perceived psychological proximity ~ distance toward concepts (Mazzuca et al., 2022) (for details, see at the OSF repository: 10.17605/OSF.IO/V8XC9).

#### Attitudes toward ecology and nature

In our experiment, we collected some information about the relationship between the two age cohorts and ecology and nature. Specifically, we asked participants about their perceived level of expertise on ecology and nature-related topics, their estimated frequency of updates on ecology-related topics, their perceived frequency of engagement in ecology-related activities (e.g., climate change activism, pro-environmental behaviors), their perceived frequency of engagement in *green* activities (e.g., gardening), their perceived frequency of physical activity (e.g., jogging) in natural outdoor environments (e.g., park), and their perceived level of passion for nature. Participants answered each question using a 7-point Likert scale, where higher scores indicated higher levels of expertise, update, engagement, and passion, while lower scores indicated the opposite.

### Procedure

The Ethics Committee of the Department of Dynamic and Clinical Psychology, and Health Studies, Sapienza University of Rome (Prot. n. 0002010—30/11/2022) granted ethics permission. The study was carried out in accordance with the Declaration of Helsinki. The main experiment included two sessions. Each participant attended both sessions in person, with the same experimenter, and within the same setting. For theoretical reasons (please, see Sects. “Introduction”, and “Experimental hypotheses”. 2—H3 and H4 hypotheses), three independent groups of participants (*n* = 16 older and *n* = 16 younger adults from each age group) were randomly assigned to one of three different quiet and lighting contexts, i.e., a room (Indoor condition), a natural outdoor setting (Natural Outdoor condition), or an urbanized outdoor setting (Urbanized Outdoor condition). Within each condition, the specific setting (e.g., Indoor: a house or laboratory’s room; Natural Outdoor: a park or a house’s garden; Urbanized Outdoor: a public square or a house’s balcony) was chosen because easily accessible for participants and it varied between them, although we took care in maintaining some constant elements differentiating the three conditions (please, see Table S4, SM).

In the first session, which lasted, on average, 15–20 min, we first required participants to provide socio-demographic information (i.e., age, sex, gender, education level, occupation, native language—for descriptive statistics, see Table S1, SM), then to perform the categorization task.

A few days later (up to two weeks), we re-contacted participants for the second session, and they completed the feature generation and then the rating task. Finally, participants provided information on the *greenness* of the places where they live and their attitudes toward ecology and nature (Sect. “Attitudes toward ecology and nature” and Table S1). The second session took, on average, 1.5 h per participant.

### Tasks

*Categorization.* We implemented the task on Psytoolkit^3^ (Stoet, 2017) and administered it individually on a laptop with a 15-inch monitor. We asked participants to keep their response finger on the spacebar and press it only when words unrelated to animals appeared, responding as soon and accurately as possible (Go/No-Go task). In each trial, a fixation cross appeared for 1000 ms, followed by a written word which remained on the screen until the response or 3000 ms without response. If participants pressed the spacebar when animal words appeared, an error message appeared. A 500-ms black screen concluded each trial (for a similar procedure, Barca et al., 2020). The experiment was preceded by a training phase (20 trials composed of 5 concepts for each category—Table S2, SM). Word presentation order was randomized within participants, and no word was used more than once.

*Rating.* We asked participants to evaluate abstract, concrete, and ecological concepts on seven semantic dimensions using 7-point Likert scales (Sect. “Semantic dimensions”) through a survey implemented on Qualtrics^4^ online platform. Dimensions were randomly presented for each participant, and target-words were randomly presented within each dimension.

*Feature Generation.* Participants performed the task within the same Qualtrics survey. We randomly presented participants with abstract, concrete, and ecological concepts and asked them to orally produce all the properties (i.e., adjectives) they deemed typically true for each concept within 1 min. We allowed participants two extra minutes if they did not provide at least three properties for a given word. We audio-recorded participants’ verbal production and manually transcribed it afterward.

## Data analysis

We used RStudio (R Core Team, 2023, version 4.3.0) to preprocess (“Tidyverse” R’s package, Wickham et al., 2019) and analyze data.

*Categorization task.* We first calculated the percentage of accurate responses, then cleaned RTs for accurate responses (“trimr” R’s package, Grange, 2022) by computing the average RTs for each participant and removing data points exceeding ± 3 *SD* from their mean (Zimmerman & Williams, 2000). Since RTs were not normally distributed, differently from what declared in the preregistration, we analyzed them through a generalized linear mixed model with a log-gamma distribution (“lme4” R’s package, Bates et al., 2015). The model featured RTs as dependent variable, Category of Word (Abstract, Concrete, Ecological), Group (Younger, Older), Setting (Indoor, Natural Outdoor, Urbanized Outdoor), and their interaction as fixed effects, and Target Words and Participants as random intercepts. The significance of fixed effects and interactions for the model was determined with Type III ANOVAs (“car” R’s package, Fox & Weisberg, 2019), and *p*-values were calculated using Wald’s Chi-squared tests.

*Rating task.* We first assessed the internal consistency of the ratings provided on each dimension by the two age cohorts, calculating Cronbach’s alphas (Cronbach, 1951) separately for each group (“psych” R’s package, Revelle, 2024). Then, to explore differences in ratings across kinds of concepts and age groups, we fitted separate mixed-effects ordinal regression models on each dimension (“ordinal” R’s package, Christensen, 2022), including rating scores as a dependent variable, the interaction between Category of Word and Group as a fixed factor, and Target Words and Participants as random intercepts. The significance of fixed effects and interactions was calculated as for RTs (but using “RVAideMemoire” R’s package, Hervé, 2022).

*Feature generation task.* We first explored differences across age groups and categories of words in the number of listed features and unique features by fitting two generalized linear mixed models with a Poisson distribution (“lme4” R’s package). The first model featured the number of features as a dependent variable, Group, Category of Word and their interaction as fixed factors, and Target Words and Participants as random intercepts. The second model was similar to the previous one but featured the number of unique features as a dependent variable and only Target Words as random intercepts. The significance of fixed effects and interactions was calculated as for RTs.

Next, to investigate generational differences in the content of ecological knowledge, we followed an established pipeline for the preprocessing and analysis of feature listing data (Falcinelli et al., 2024b; Mazzuca et al., 2022). We first identified—for reasons of conciseness—the most representative ecological concept (the one with the highest representativeness score—Sect. “Words”), and we manually pre-processed related associations, e.g., correcting mistakes and typos, unifying synonyms of the same word and morphological variations of the same root, i.e., singular/plural and masculine/feminine forms.

Then, to explore the content of listed features, we performed qualitative analyses, separately for older and younger adults, investigating the most important topics encompassed by (1) the most salient features; (2) the communities (i.e., clusters of features) composing semantic networks of associates.^5^

We extracted the most salient features by identifying those generated by at least 10% of participants and calculating their cognitive salience index, a measure of their relevance within all generated associations (Sutrop, 2001).^6^

To extract communities, we first created two age-separate undirected weighted semantic networks on associations (“igraph”, Csardi & Nepusz, 2006, “tidygraph”, Pedersen, 2019, and “ggraph”, Pedersen, 2020, R’s packages), using the count of co-occurrences of bigrams (i.e., pairs of features listed in succession—“tidytext” R’s package, Silge & Robinson, 2016) as input. We then detected communities through a Louvain’s algorithm (Blondel et al., 2008) and visualized the networks using a Fruchterman–Reingold force-directed layout algorithm (Fruchterman & Reingold, 1991).

*Attitudes toward ecology and nature across age groups.* Differently from what we declared in the preregistration plan (10.17605/OSF.IO/CV3E2), we assessed *a-priori* differences in ecology and nature-related attitudes between age groups by running separate separate mixed-effects ordinal regression models on each characteristic instead of independent t-tests, since the former better fitted with the kind of data under scrutiny (i.e., Likert scale data—Taylor et al., 2023). All models featured rating scores for the characteristic of interest as a dependent variable, Group as a fixed factor, and Participants as random intercepts (“ordinal” R’s package). The significance of the main effects was determined through Type II ANOVAs, with *p*-values calculated using Wald’s Chi-squared tests (“RVAideMemoire” R’s package).

*Post hoc comparisons and marginal R*^*2*^* for statistical models.* For all statistical models, comparisons across conditions were performed with Tukey’s adjustments (“emmeans” R’s package, Lenth, 2023).

## Results

In the subsequent sections, we will present results we gained from the three tasks (5.1—5.3) along with assessing potential differences across age groups in their ecology and nature-related attitudes (5.4). Out of curiosity, we also performed some supplementary analyses to explore whether these attitudes might have influenced participants’ performances for ecological concepts across the three tasks (please, see Online Appendix B, SM).

### Categorization task

Given the simplicity and brevity of the task, the overall level of response accuracy was high (Correct answers = 99%).

In line with our expectations, the main model on RTs showed a main effect of Group, ${\chi }^{2}$(1) = 10.1959, *p* < 0.0001, and a main effect of Category of Word, ${\chi }^{2}$(2) = 19.5607, *p* < 0.0001, but contrary to our expectations, it did not yield a main effect of Setting, ${\chi }^{2}$(2) = 2.6680, *p* = 0.263, nor a significant interaction between Group and Setting, ${\chi }^{2}$(2) = 3.0815,* p* = 0.214.

**Fig. 1:**
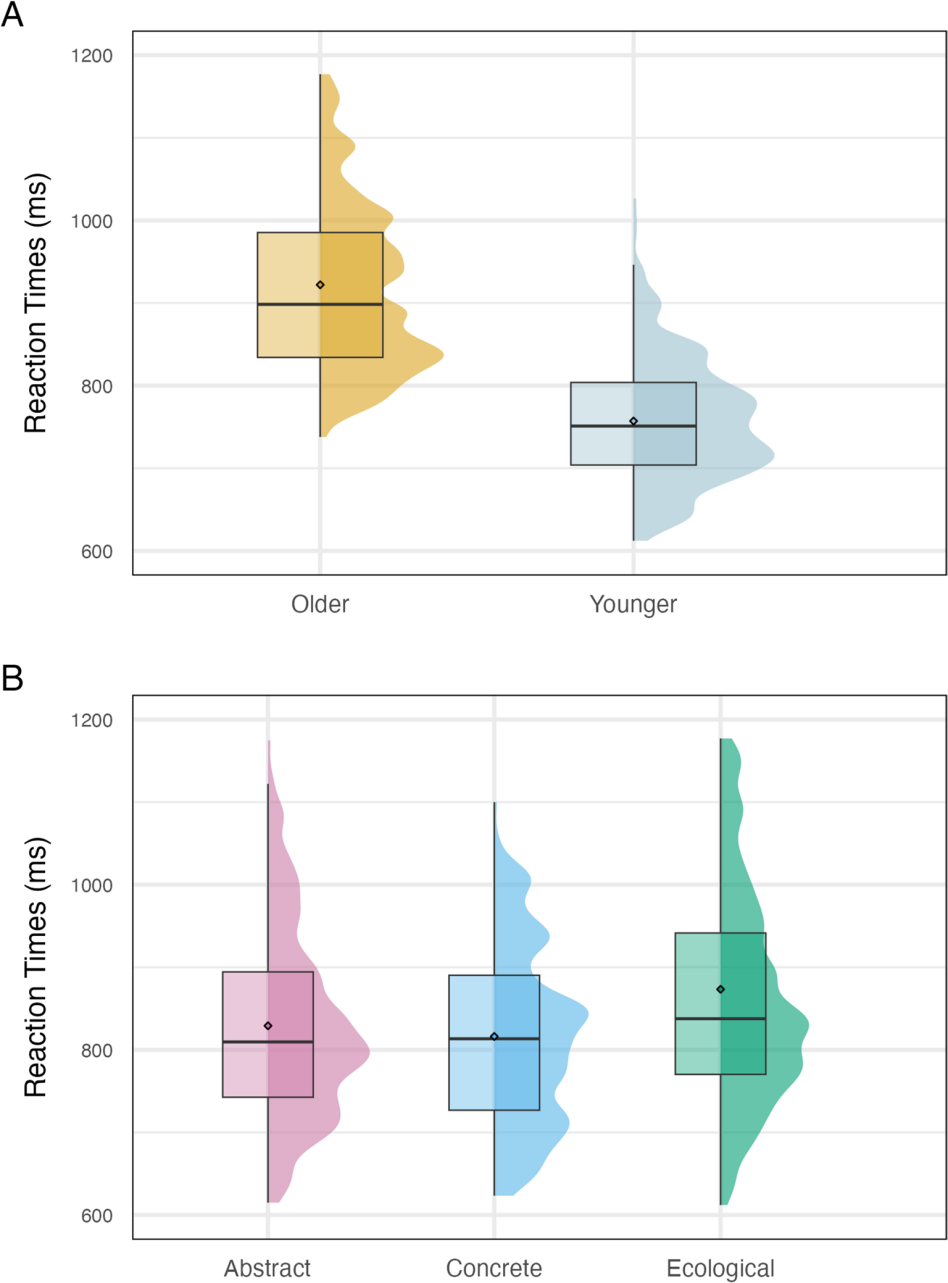
(Distribution of) reaction times shown by older and younger participants for all critical words (Panel **A**) and by the whole sample for abstract, concrete, and ecological concepts (Panel **B**). In the boxplots, black circles indicate the mean value, bold black horizontal lines the median, boxplot’s vertical extremes represent the data’s minimum and maximum values, while their height the interquartile range (upper side: 75th—bottom side: 25th percentile)

Older adults showed slower RTs than younger adults, *z* = 3.310, d = 0.94, *p* < 0.001 (Fig. 1, Panel A; Table S5, SM, reports descriptive statistics of RTs).

Ecological concepts were processed slower than both abstract, *z* = 2.865, d = 0.251, *p* = 0.012, and concrete concepts, *z* = 3.342, d = 0.293,* p* = 0.002, and we did not find differences in RTs between abstract and concrete concepts, *z* = 0.479, d = 0.042, *p* = .881 (Fig. 1, Panel B) (for a replication of results with another type of filler words, Online Appendix A, SM).

### Rating task

The interrater reliability was excellent for both age groups: Cronbach’s alphas ranged from .87 to .95 for the older sample and from .89 to .95 for the younger sample (Table S6, SM).

All statistical models returned a significant interaction between Category of Word and Group, all *p*_*s*_ < 0.020. Below, we schematically illustrate results for each dimension, while Table 1 reports statistical details from comparisons (for a summary of results, Table S7, SM).

**Table 1:** Details from statistical models performed on each semantic dimension. In the table, the second column reports the targeted dimension, the third column shows results (degree of freedom, Likelihood Ratio Chi-square value, and *p*-value) of the interaction between Category of Word and Group from the ANOVA models, while the fourth column shows results from pairwise post hoc comparisons across conditions (z-value, standard error and *p*-value). In the fourth column, the “ < ” and “ > ” signs indicate that the first term received a statistically significant lower and higher score, respectively, than the second term, while the equal sign (“ = ”) indicates that the score to the two terms did not statistically differ

Panel	Dimension	Category of word * group			Results from pairwise comparisons (*z,* SE*, p*)
		df	*X*^*2*^ value	*p*-value	
A	Concreteness ~ abstractness (level of abstractness)	2	7.80	0.020	Ecological older = ecological younger (1.51, 0.235, 0.132)
					Ecological < abstract (older: − 6.55, 0.385, < 0.0001; younger: -7.75, 0.386, < 0.0001)
					Ecological > concrete (older: 9.90, 0.408, < 0.0001; younger: 9.20, 0.410, < 0.0001)
B	Age of acquisition (level of late acquisition)	2	85.36	< 0.0001	Ecological older > ecological younger (5.65, 0.267, < 0.0001)
					Ecological > concrete (older: 9.87, 0.626, < 0.0001; younger: 7.46, 0.620, < 0.0001)
					Ecological older > abstract (2.98, 0.616, 0.008)
					Ecological younger = abstract (0.96, 0.613, 0.605)
C	Familiarity (level of familiarity)	2	44.14	< 0.0001	Ecological older = ecological younger (1.76, 0.287, 0.079)
					Ecological = abstract (older: 0.93, 0.377, 0.622; younger: 0.90, 0.374, 0.639)
					Ecological < concrete (older: − 7.37, 0.394, < 0.0001; younger: − 4.49, 0.380, < 0.0001)
D	Social metacognition (level of need of the others’ help to understand the concept’s meaning)	2	16.65	< 0.001	Ecological = abstract (older: − 0.53, 0.341, 0.855; younger: − 1.73, 0.342, 0.196)
					Ecological > concrete (older: 12.54, .374, < .0001; younger: 10.69, .356, < .0001)
					Ecological older = ecological younger (-1.80, .374, .072)
E	Word confidence (level of confidence in mastering the concept’s meaning)	2	8.09	0.018	Ecological = abstract (older: 0.89, 0.272, 0.649; younger: 1.71, 0.270, 0.202)
					Ecological < concrete (older: − 8.99, 0.291, < 0.0001; younger: − 7.30, 0.279, < 0.0001)
					Ecological older > ecological younger (2.24, 0.323, 0.025)
F	Openness to negotiation (level of openness to negotiate the conceptual meaning)	2	26.48	< 0.0001	Ecological older = ecological younger ( 0.41, 0.372, 0.679)
					Ecological < abstract (older: − 2.52, 0.253, 0.032; younger: − 3.13, 0.250, 0.005)
					Ecological > concrete (older: 14.31, 0.272, < 0.0001; younger: 11.30, 0.260, < 0.0001)
G	Perceived distance (level of psychological distance felt from the concept)	2	20.66	< 0.0001	Ecological older < ecological younger (− 6.12, 0.249, < 0.0001)
					Ecological < abstract (older: − 5.18, 0.197, < 0.0001; younger: − 2.56, 0.195, 0.028)
					Ecological > concrete (older: 4.88, 0.206, < 0.0001; younger: 3.83, 0.197, < 0.001)

*Concreteness* ~ *Abstractness.* The level of abstractness attributed to ecological concepts did not significantly differ between older and younger adults. Both age groups evaluated ecological concepts as less abstract than abstract concepts but more abstract than concrete ones (Table 1, Panel A).

*Age of Acquisition.* Older adults judged to have acquired ecological concepts later than younger participants. Older adults rated to have acquired ecological concepts significantly later than abstract concepts, while younger adults at a similar age as abstract concepts. Both age groups rated ecological concepts as acquired significantly later than concrete concepts (Table 1, Panel B).

*Familiarity*. Older and younger adults perceived ecological concepts similarly familiar. Both age groups judged ecological concepts as familiar as abstract concepts and less than concrete concepts (Table 1, Panel C).

*Social Metacognition.* Older and younger adults estimated to need others to understand ecological concepts’ meaning to a similar extent between each other and abstract concepts, and more than for concrete concepts (Table 1, Panel D).

*Word Confidence*. Older adults perceived they mastered ecological concepts’ meaning more than younger adults. Both age groups judged to master the meaning of ecological concepts to a similar extent as abstract concepts and less than concrete concepts (Table 1, Panel E).

*Openness to Negotiation*. Older and younger adults reported to be similarly open to negotiate the meaning of ecological concepts, less than abstract ones but more than concrete concepts (Table 1, Panel F).

*Perceived Distance*. Older adults perceived ecological concepts psychologically closer than younger adults. Both age groups judged ecological concepts as psychologically closer than abstract concepts but farther than concrete concepts (Table 1, Panel G).

### Feature generation task

#### Number of listed and unique features across conceptual kinds

Both models on the number of features and unique features yielded a main effect of Category of Word (listed features: ${\chi }^{2}$(2) = 20.8638, *p* < 0.0001; unique features: ${\chi }^{2}$(2) = 11.8284, *p* = 0.003), but not a significant interaction between Category of Word and Group (listed features: ${\chi }^{2}$(2) = 1.6109, *p* = 0.447; unique features: ${\chi }^{2}$(2) = 2.4949, *p* = 0.290).

**Fig. 2:**
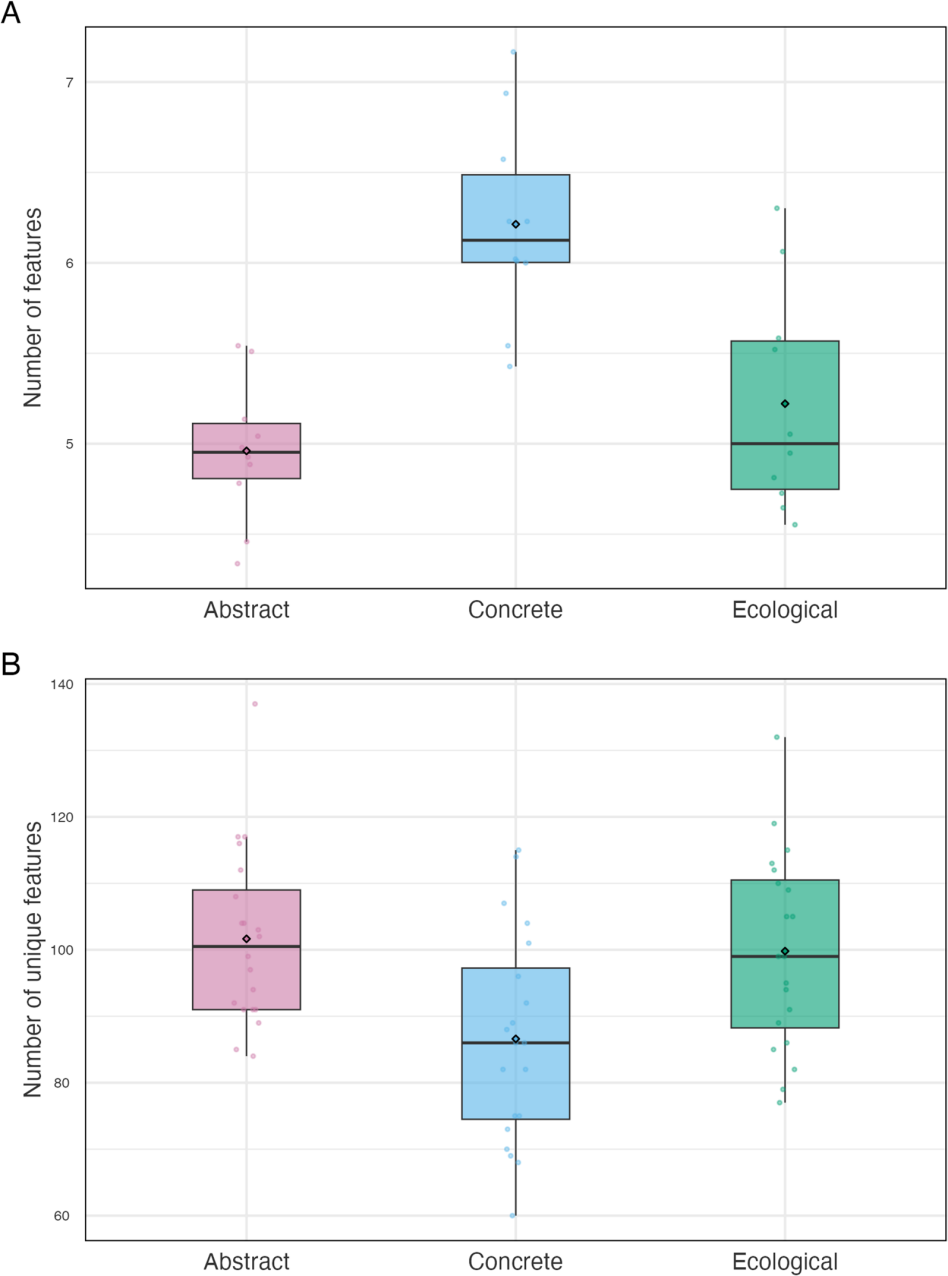
Number of features (Panel **A**) and unique features (Panel **B**) generated by the whole sample for abstract, concrete and ecological concepts. In the boxplots, black circles indicate the mean value, bold black horizontal lines the median, boxplot’s vertical extremes represent the data’s minimum and maximum values, while their height the interquartile range (upper side: 75th—bottom side: 25th percentile). Colored dots represent data points

Both older and younger individuals listed significantly fewer associations and more unique features for both ecological (listed features: *z* = − 4.259, SE = 0.049, *p* = 0.0001; unique features: *z* = 3.085, SE = 0.040, *p* = 0.006), and abstract concepts (listed features: *z* = 5.394, SE = 0.033, *p* < 0.0001; unique features: *z* = 3.590, SE = 0.054, *p* = 0.001) than concrete concepts. Importantly, we found no differences between ecological and abstract concepts in both these aspects (listed features: *z* = − 1.136, SE = 0.040, *p* = 0.492; unique features: *z* = − 1.136, SE = 0.040, *p* = 0.492) (Fig. 2).

#### The semantic representation of “Recycling” across generations.

The most representative word among ecological ones was “Recycling” (Italian: “Riciclo”; *M representativeness* = 6.52).

All participants produced for this target word 456 associations (*M* = 4.75; *SD* = 2.07, 214 different features), with older adults generating 197 associations (*M* = 4.10; *SD* = 1.26, 116 different features) and younger adults 259 associations (*M* = 5.40; *SD* = 2.50, 140 different features).

#### The content of most salient features

The number of most frequently generated associates was similar between older and younger participants (six features overcoming the 10% threshold for older, seven for younger adults; Table 2). The word “*Useful*” was the most frequently generated and the only feature shared by the two groups.

**Table 2:** Most frequent terms for “Recycling” produced by at least 10% of older and younger adults, their English translation, their mean rank, the percentage of participants producing each term along with the raw frequency, and their Cognitive Salience Index. Words in bold represent adjectives shared between the groups

Italian word	English translation	Mean rank	Percentage of participants producing the feature (raw frequency)	Cognitive salience index
*Older adults*				
**Utile**	**Useful**	3.59	35.42 (17)	0.10
Economico	Economic	3.63	16.67 (8)	0.05
Ecologico	Ecological	3.67	12.50 (6)	0.03
Materiale	Material	3.00	10.42 (5)	0.03
Ottimo	Excellent	3.80	10.42 (5)	0.03
Riutilizzato	Reused	2.60	10.42 (5)	0.04
*Younger adults*				
**Utile**	**Useful**	3.37	39.58 (19)	0.12
Ambientale	Environmental	3.27	22.92 (11)	0.07
Giusto	Right	3.40	20.83 (10)	0.06
Pulito	Clean	4.20	20.83 (10)	0.05
Necessario	Necessary	3.71	14.58 (7)	0.04
Riutilizzabile	Reusable	6.60	10.42 (5)	0.02
Sostenibile	Sustainable	2.00	10.42 (5)	0.05

Most salient features differed across age groups, but not substantially: they referred to the same thematic spheres but had specific age-nuances. For example, both groups emphasized the aspect of reuse that recycling brings, but while older participants most frequently produced “*Reused*”, thus highlighting something that usually happens, younger adults most listed “*Reusable*”, remarking more the possibility than the effective action of reusing. Similarly, both samples emphasized the positive valence of recycling, but older adults more often provided a value judgment, i.e., “*Excellent*”, and younger adults a moral judgment, i.e., “*Right*”. Other associations were instead peculiar to each age group. For instance, older adults more frequently mentioned concrete advantages of recycling, i.e., “*Economic*” and “*Material*”, younger adults stressed its positive outcomes, i.e., “*Clean*” and “*Sustainable*”.

#### The content of networks’ communities

Overall, older and younger participants did not consistently differ in the number of communities and associates their semantic networks comprised, although features were slightly less interconnected in the older network. The older adults’ network comprised 116 nodes, 141 edges and 13 main communities; the younger adults’ network 140 nodes, 207 edges and 11 main communities.

**Fig. 3:**
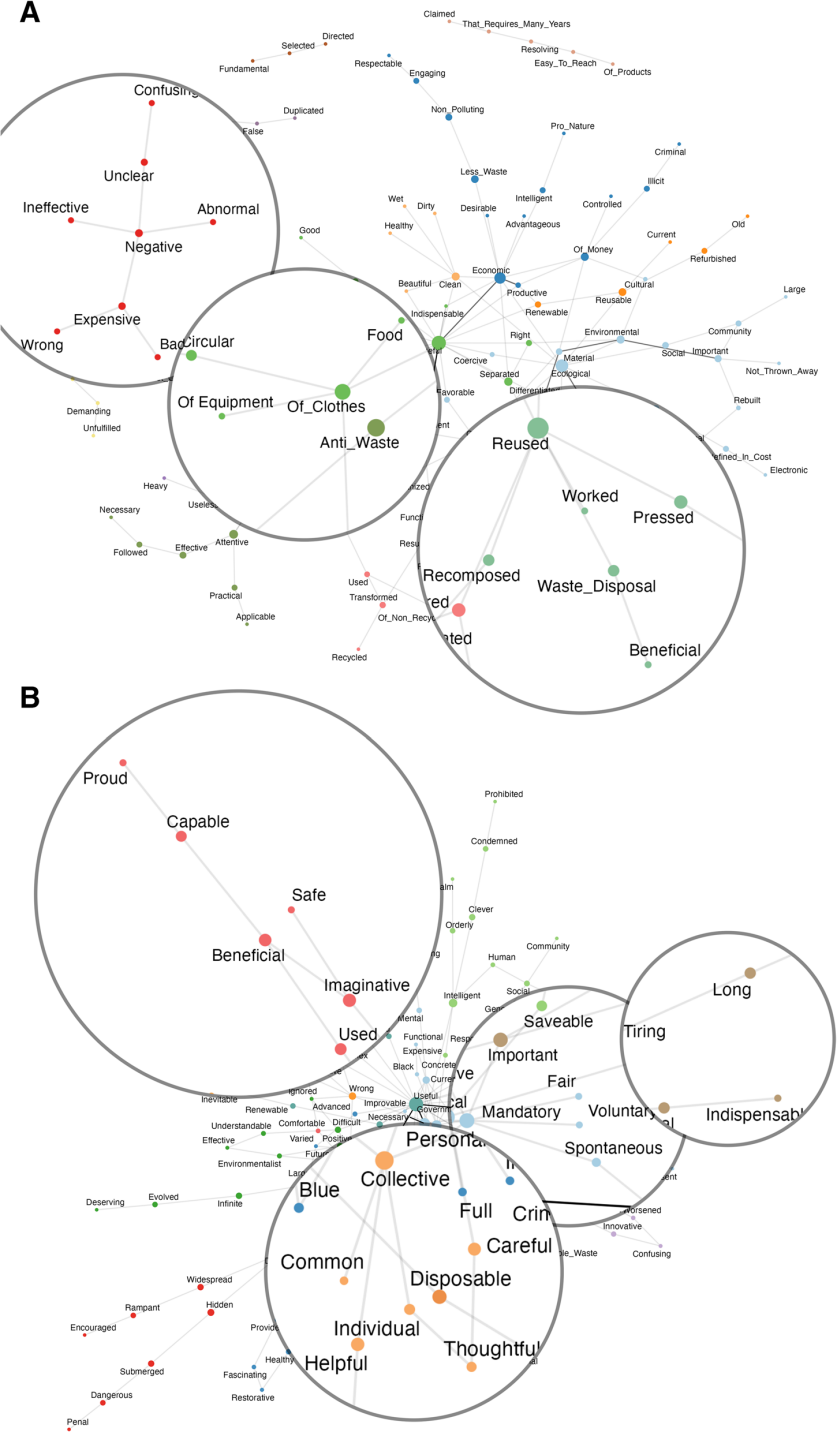
Network of features listed by older (Panel **A**) and younger adults (Panel **B**) for “Recycling”. In the graphs, we zoomed only on communities discussed in the main text. Figures without zooming can be found in Online Appendix C, SM

In line with previous results, the semantic representation of “Recycling” shared both similarities and differences across age groups. For example, both older and younger adults’ networks encompassed features including both positive and negative aspects of recycling. However, older adults more often (i.e., through more communities) emphasized negative aspects of “recycling” like its inefficiency and lack of organization (e.g., red community, Fig. 3, Panel A), while younger adults more frequently stressed its positive sides like its efficiency and utility (e.g., red community, Fig. 3, Panel B) and referred to an opposition between individual and societal benefits (e.g., orange community in Fig. 3, Panel A).

Unlike younger adults, older adults emphasized more the aspect of recycling related to reusing and giving new life to objects (e.g., green community, Fig. 3, Panel A) and listed more frequently the kinds of entities that can be recycled (e.g., light green community, Fig. 3, Panel B). Conversely, younger adults stressed more the obligatory/voluntary character of recycling (e.g., light blue community, Fig. 3, Panel B), its importance and the efforts it requires (e.g., brown community, Fig. 3, Panel B) (for an extended discussion, Online Appendix C, SM).

### Differences between age groups in their ecology and nature-related attitudes

Older and younger participants significantly differed in their attitudes toward ecology and nature. In particular, the two cohorts were significantly different in their level of expertise on natural topics, ${\chi }^{2}$(1) = 7.4896, *p* = 0.006, with older participants perceiving themselves as more expert than younger participants, b = 1.0352, SE = 0.443, *z* = 2.449; in the frequency of updates on ecology-related topics, ${\chi }^{2}$(1) = 6.4958, *p* = 0.011, with older participants reporting more frequent updates on ecology, b = 0.9918, SE = 0.432, *z* = 2.295; in their level of engagement in ecology-related activities, ${\chi }^{2}$(1) = 15.131, *p* < 0.0001, and in *green* activities, ${\chi }^{2}$(1) = 25.83, *p* < 0.0001, with older adults more frequently committed in ecology-related, b = 1.5541, SE = 0.486, *z* = 3.199, and nature-related habits, b = 1.9773, SE = 0.409, *z* = 4.838; and in their passion toward nature, ${\chi }^{2}$(1) = 8.2553, *p* = 0.004, with older adults more passionate than younger adults, b = 1.1449, SE = 0.281, *z* = 4.072. Instead, we found no differences across age groups in their level of expertise on ecology-related topics, ${\chi }^{2}$(1) = 1.2649, *p* = 0.260, and their frequency of physical activity in natural outdoor environments, ${\chi }^{2}$(1) = 0.53732, *p* = 0.464 (for descriptive statistics, Table S1, SM).

## Discussion

Results provide insights into ecological conceptualization, addressing generational differences across multiple data.

As for conceptual processing (categorization task, Sect. “Categorization task”), older participants were slower in categorizing than younger participants, in keeping with our H1 hypothesis (Hultsch et al., 2002; Lucci et al., 2013; see also Falcinelli et al., 2024b).

In line with the H2.3 hypothesis, older and younger participants processed ecological concepts significantly slower than abstract and concrete concepts. We did not find the classical Concreteness Effect in neither age groups. Its absence may be due to the filler words, i.e., animal concepts. Indeed, the Concreteness Effect was present in the control categorization task (Online Appendix A, SM), where filler words were astrological entities instead of animals. Inserting animals, usually conceived as concrete concepts, might have interfered with the processing of concrete concepts (artifacts), thus obscuring this effect (Online Appendix A, SM; for similar results, Falcinelli et al., 2024b). Finally, contrary to our expectations, there were no processing differences between experimental settings (H3 hypothesis) or between older and younger participants in different contexts (H4 hypothesis). Hence, the context overall, and the exposition to a natural context, did not differently modulate categorization. To our knowledge, our study represents one of the first attempts to verify the impact of the natural setting on categorization. Most studies concern its positive influence on attention, although the results are not unanimous (Sect. “Introduction”). Exposure to a natural context may not be sufficient to improve categorization performance for at least two reasons. First, unlike other cognitive processes, the conceptual processing of known concepts may be rather impermeable to the influence of context. Second, the context may play a role, but we were unable to capture it due to methodological limitations. One is the absence of a rigorous manipulation of the settings and their variability (Sect. “Study’s limitation” for details). In this study, we intentionally adopted an ecological approach to investigate conceptual categorization, preserving the complexity of the scenarios. However, we did not control for the amount or type of multisensory stimulation within each context.

As to semantic organization (rating task, Sect. “Rating task”), it did not consistently differ between age groups (contrary to H6 and H7 hypotheses). Indeed, both older and younger adults rated ecological concepts for some aspects strictly similar to abstract concepts and with a more abstract pattern than concrete ones (in line with H5.2 hypothesis), i.e., more familiar, more mastered, and needing more the help of others to be understood than concrete concepts. For other aspects, both age groups evaluated them at the edge between abstract and concrete concepts (in line with H5.1 hypothesis), i.e., less abstract, psychologically closer, and less negotiable in meaning than abstract concepts but more abstract, psychologically farther, and more negotiable than concrete ones.

Although ecological concepts appear at the edge between abstract and concrete concepts in their abstractness score, their semantic characterization leans more toward abstractness.

In addition, contrary to our expectations (H6 and H7 hypotheses), sometimes older adults characterized ecological concepts less abstractly (i.e., more concretely) than younger adults: they evaluated ecological concepts psychologically closer and more mastered—properties usually related to concreteness (Leviston et al., 2014; Mazzuca et al., 2022).

Results on conceptual representation, expressed by feature retrieval, confirmed the more abstract characterization of ecological concepts in both age groups (Sect. “Number of listed and unique features across conceptual kinds”). Older and younger adults generated a similar number of features and unique features for ecological and abstract concepts and significantly fewer properties and more unique features for them than for concrete concepts (in line with H9.2 hypothesis and replicating Canessa et al., 2021). The data on unique features are particularly interesting since it attests that independently from age, ecological concepts might have a “weaker semantic core”, being more variable across individuals than concrete concepts, as typically abstract concepts are (Borghi, 2022; Borghi & Mazzuca, 2023; Wang & Bi, 2021).

Integrating these findings, by zooming on the content of knowledge related to the ecological domain, specifically to “Recycling” target word (i.e., the most representative concept within our wordpool), results revealed that older participants did not have a less complex, diversified, and experience-driven representation than younger adults, as we expected (H8.1 hypothesis). Conversely, the knowledge associated with “Recycling”, although sharing common elements, was also differently organized across groups (in line with H8.2 hypothesis). By looking at the most salient listed features, older and younger participants in most cases emphasized similar aspects, enriched with some age-idiosyncratic nuances—for instance, both age groups characterized recycling as something right, but while older adults provided to it a value judgment (*Excellent*), younger adults a moral one (*Right*). For other substantial aspects, the most salient features of the two groups differed. For instance, recycling elicited more thoughts about recycled objects in older adults, about its positive outcomes in younger adults. Similarly, older and younger semantic networks of associates did not significantly diverge in content but exhibited age differences. Older adults focused more on reuse and recyclable entities, while younger adults emphasized more the obligatory/voluntary character of recycling, its importance, and the effort it requires. Overall, these findings are consistent with literature showing minimal differences in the content of ecology-related knowledge across the lifespan (Corner et al., 2015).

Finally, we found significant differences between older and younger adults in their ecology and nature-related attitudes (H10 hypothesis). First, older adults showed more positive attitudes toward ecology than younger ones, describing themselves as more frequently updated on ecology-related topics and more often engaged in ecology-related activities. These results do not entirely align with previous literature suggesting higher scores in such attitudes at a younger life-stage (Ballew et al., 2020; Wiernik et al., 2013). However, they are still informative as they may contribute to broadening the existing literature on the relationship between older adults and climate change, which is overlooked and sometimes controversial (Ayalon et al., 2023). Second, in line with previous evidence (Scott et al., 2015), older adults exhibited a more positive attitude toward nature than younger adults, perceiving themselves as more expert on nature-related topics, more frequently committed to nature-related habits, and more passionate about nature than younger adults. Although these attitudes did not significantly impact the performances with ecological concepts in the three tasks (Online Appendix B, SM), the higher level of experiences with ecology and nature in older adults might explain their more concrete characterization of ecological concepts in the rating task, based on previous evidence showing a positive link between expertise/experiences with a domain and its level of concreteness (Mazzuca et al., 2025a, 2025b; Villani et al., 2022). In addition, it might also elucidate some peculiarities in older adults’ verbal production for “Recycling” target word—e.g., both older and younger adults related recycling to the practice of reusing, but while older adults spoke of reuse as something that is usually done, younger adults spoke of it as something that might happen.

Overall, our study provides evidence that conceptual categorization is a multifaceted process, influenced by a network of interconnected factors including generational differences and life experiences.

### Study’s limitations

This study is not free from limitations that future research may address. Firstly, our sampling method relied on snowballing, a convenience-based procedure that may limit the representativeness of our findings. Secondly, to be consistent and provide a good extension of the existing Western literature on the relationship between different age groups and climate change (Sect. “Introduction”), we tested only Italian younger and older adults. However, further insights might come from comparing more fine-grained distinct age cohorts that cover the entire lifespan (e.g., children, adolescents, younger adults, middle-aged adults, older adults, centenarians) as well as people from different Western and East nationalities. Indeed, this might help strengthen the generalizability of our results. Lastly, our study primarily focused on age-group differences in ecological and nature-related attitudes (Sect. “Attitudes toward ecology and nature”). Consequently, we didn’t collect detailed information on participants’ actual pro-environmental behaviors, apart from a general index about the estimated level of engagement of participants with ecology-related activities (Sect. “Attitudes toward ecology and nature”). Hence, future studies might better address this aspect by examining whether and how participants’ actual pro-environmental behaviors (but also the environmental quality—greenness/pollution—of their living environments) may impact the categorization of ecological phenomena.

Concerning the setting, as previously noted (Sect. “Discussion”), one methodological limitation was the need to sacrifice methodological rigor in favor of a more ecological approach. In addition, the settings were also chosen based on a convenience criterion for participants (e.g., indoor/natural/urbanized places that were easily accessible for them—see Sect. “Procedure”), thus making the specific testing places differ within each condition (e.g., a garden or a park for the natural outdoor condition—Sect. “Procedure”), although the presence of some constant elements within each of them (Table S4, SM). These choices prevented us from rigorously controlling for the multisensory stimulation that participants experienced during the experimental session. The lack of control over these potential confounding variables may have been decisive in preventing context-dependent effects from emerging during conceptual processing.

In addition, although our experimental procedure had the strength to include the co-presence of participant and experimenter, thus avoiding the nowadays frequent reliance on online data (“Mturkification”, Anderson et al., 2019; review by Tam et al., 2021) and allowing to support older adults with the tasks, we cannot exclude an effect of the experimenter’s influence over the participants’ performance (Yiping, 2025).

Concerning the tasks, it would be worthwhile for future research to exploit other indexes from the feature generation data to shed more light on the hypothesized *hybrid* nature of ecological concepts—e.g., examining the type of features and their listing order. Indeed, people usually produce more and earlier perceptual features for concrete than abstract concepts (Breedin et al., 1994; Canessa et al., 2021). Considering these parameters could further allow testing whether ecological concepts are more similar to abstract or concrete ones and verify whether and how this impacts pro-environmental behaviors.

## Conclusion and study’s implications

This is the first study addressing ecological concepts through the lens of abstractness by targeting different aspects—processing, representation, and organization. We found that ecological concepts are, overall, more similar to abstract concepts (mainly in their semantic organization and representation) or more abstractly characterized than both abstract and concrete concepts (mainly in conceptual processing). There were no stark differences between older and younger participants, but some age-dependent nuances, especially in the knowledge content and semantic organization, with older adults—who showed more positive eco-attitudes—having a more concrete representation of the ecological domain than younger people.

From a theoretical side, these results support the flexibility of our conceptual system (Borghi, 2023). First, they show that ecological concepts present some characteristics that go beyond the traditional *concrete–abstract* dichotomy. Indeed, although being more similar to abstract than concrete concepts, they also possess some specific peculiarities (such as their *over-abstract* characterization). In this sense, our findings align with recent theories proposing to overcome this rigid distinction (Barsalou et al., 2018; Borghi et al., 2017; Banks et al., 2023), since it cannot fully explain differences in conceptualizing sub-domains, especially if new as ecology (Mazzuca et al., 2025a, 2025b).

Second, our results extend previous evidence on the multifarious nature of ecological concepts (Falcinelli et al., 2024a), by showing that their level of abstractness can also vary depending on age. In this sense, they converge with Barsalou et al.’s (2018) proposal that concepts should be categorized within situated situations. In this view, individuals process elements in relation to their personal characteristics, such as age, personal attitudes (Sect. “Differences between age groups in their ecology and nature-related attitudes”), motivations, goals, and the specific context in which they are embedded. Thus, conceptual categorization requires a flexible and dynamic mapping of meanings, which is grounded in spatiotemporal contexts and the continually evolving demographic peculiarities of individuals.

From a societal perspective, the fact that both younger and older adults conceptualize ecological issues abstractly, yet older adults represent climate change more concretely and show more positive attitudes, may have important practical implications for future action. Campaigns and policies often focus on younger generations, but our results suggest a more nuanced, age-specific approach is needed. To engage younger people, environmental messaging could move beyond distant, abstract threats and instead highlight immediate, tangible local impacts, like air quality or local water supply, using digital platforms they already inhabit. For older adults, who already have a more concrete understanding and positive attitudes, campaigns could focus on leveraging their existing connection to the environment. This involves emphasizing how local conservation efforts directly improve their neighborhoods or community spaces and using traditional media to reach them. Beyond campaigns, these insights can inform policy and education. For younger people, environmental education could incorporate hands-on projects, like community gardening or local clean-ups, to build a more concrete understanding. For older adults, policy can empower them as community leaders in local environmental initiatives. Ultimately, these generational differences are not a source of division but an opportunity for collaboration. By focusing on shared, local goals, we can bridge the gap between the abstract environmental concerns of the young and the lived environmental experience of the old, fostering collective action that leads to more effective and sustainable behaviors across all age groups.

## Supplementary Information

Supplementary Material 1.

## Data Availability

All materials, raw data, and analysis scripts are available at the following OSF repository: 10.17605/OSF.IO/V8XC9.s

## Footnotes

1 In the whole text, examples of concepts expressed through words will be given in italic.

2 These are all the main hypotheses of the preregistration plan, except the H9 and H10 hypotheses that we moved from exploratory to main, detailing them before the data analysis.

3 https://www.psytoolkit.org/

4 https://www.qualtrics.com/it/

5 Semantic networks are knowledge structures showing how category exemplars are related. Nodes represent exemplars (e.g., *functioning,* *chaotic* for the “Recycling” concept), and edges indicate the likelihood of one being generated when another is mentioned (Cosgrove et al., 2021).

6 The Cognitive Salience Index, ranging from 0 to 1, reflects a term’s importance in free-listing tasks by combining its frequency and average position. It is calculated as follows: CS = F/(NmP), where F = term frequency, N = number of participants, and mP = mean position of the term (Sutrop, 2001; van Putten et al., 2020).
