# Supplementary material for: Thinking climate change through the lens of abstractness: a multi-task and multi-setting investigation into generational differences in the conceptualization of ecology
Source: Cogn Res Princ Implic. 2025 Nov 16;10:79. doi: 10.1186/s41235-025-00689-4 (PMC12620345; doi:10.1186/s41235-025-00689-4)
Supplement: Supplementary file 1 — Supplementary Material 1. [file 41235_2025_689_MOESM1_ESM.docx]

**Supplementary Materials of the paper “Thinking Climate Change through the Lens of Abstractness: a Multi-Task and Multi-Setting Investigation into Generational Differences in the Conceptualization of Ecology”.**

***Appendix A.*** *“Control” categorization task study*

To be sure that results we obtained on the processing disadvantage of ecological over abstract and concrete concepts (section 5.1) could be attributed to their intrinsic nature rather than to a possible interference effect due to animal filler words—indeed, both ecological and animal are *green* concepts, and some ecological concepts in our wordpool implicitly referred to animals (e.g., *fauna, natural reserve*); thus, this might have caused an interference effect making RTs for ecological concepts shower—, and with the broader aim to provide robustness to our main findings, we conducted a further non-preregistered “control” categorization task on an independent sample of older and younger individuals.

The experiment’s structure, materials and methods were identical to the main study (section 3), with the only difference that in this case filler words consisted of astrological concepts (e.g., *spaceship, galaxy, meteor*; for an overview of the experimental stimuli, Table S2, SM). We chose this category since it included words indicating non-*green* concepts that were not semantically related to any of our critical words (abstract, concrete, ecological). Similarly to animal concepts (section 3.2.1), we selected astrological words through consultation among ourselves.

On a procedural side, we tested three independent groups of participants (*n* = 4 older and *n* = 4 younger adults from each age group) in one of the three possible settings (section 3.3). We first asked them to provide the same socio-demographic information as at the beginning of our main experiment (section 3.3) and then to perform the “control” categorization task. Finally, participants provided information on the *greenness* of the places where they live and their attitudes towards ecology and nature, as in the last part of our main experiment (section 3.3). The procedure took, on average, 20/25 minutes per participant.

Statistical analyses were the same as for the main study (section 4). Materials, raw data, and analysis scripts can be found at the OSF repository (https://doi.org/10.17605/OSF.IO/V8XC9).

Below, we report information on the sample, the task’s results, and a brief discussion of them.

*Participants.* Since this study represented just a “control” follow-up, we tested half of the original sample for each experimental condition. The sample was therefore composed of 48 participants, i.e., 24 older (13 females, *M_age_* = 73.25; *SD_age_* = 4.35; *Range_age_* = 66 - 81) and 24 younger adults (19 females, *M_age_* = 26.83; *SD_age_* = 4.77; *Range_age_* = 19 - 33), recruited through word-of-mouth. Similarly to the main sample, the two age cohorts met all the study inclusion criteria (section 3.1; MMSE of the older sample: *M* = 27.30; *SD* = 2.36). Table A.1 summarizes socio-demographic information of the sample.

***Table A.1.*** *Socio-demographic information of participants of the “control” categorization task study.*

| **Baseline characteristics** | **Group** | **Descriptives** | |
| --- | --- | --- | --- |
|  |  | **n** | **%** |
| Sex |  |  |  |
| Male | Older | 11 | 23 |
|  | Younger | 5 | 10 |
| Female | Older | 13 | 27 |
|  | Younger | 19 | 40 |
| Intersex | Older | 0 | 0 |
|  | Younger | 0 | 0 |
| Gender |  |  |  |
| Men | Older | 11 | 23 |
|  | Younger | 5 | 10 |
| Woman | Older | 13 | 27 |
|  | Younger | 19 | 40 |
| Genderqueer | Older | 0 | 0 |
|  | Younger | 0 | 0 |
| Non-binary | Older | 0 | 0 |
|  | Younger | 0 | 0 |
| Other | Older | 0 | 0 |
|  | Younger | 0 | 0 |
| Title of study |  |  |  |
| Elementary school diploma | Older | 1 | 2 |
|  | Younger | 0 | 0 |
| Middle school diploma | Older | 9 | 19 |
|  | Younger | 1 | 2 |
| High school diploma | Older | 7 | 15 |
|  | Younger | 17 | 35 |
| Bachelor’s and/or Master’s degree | Older | 6 | 13 |
|  | Younger | 4 | 8 |
| Post-lauream title (master, PhD, etc.) | Older | 1 | 2 |
|  | Younger | 2 | 4 |
| Profession |  |  |  |
| Employed | Older | 2 | 4 |
|  | Younger | 18 | 38 |
| Student | Older | 0 | 0 |
|  | Younger | 4 | 8 |
| Unemployed | Older | 0 | 0 |
|  | Younger | 2 | 4 |
| Retired | Older | 22 | 46 |
|  | Younger | 0 | 0 |
| Socio-economic status |  |  |  |
| Extremely low income | Older | 0 | 0 |
|  | Younger | 0 | 0 |
| Low income | Older | 4 | 8 |
|  | Younger | 5 | 10 |
| Middle income | Older | 20 | 42 |
|  | Younger | 19 | 40 |
| High income | Older | 0 | 0 |
|  | Younger | 0 | 0 |
| Extremely high income | Older | 0 | 0 |
|  | Younger | 0 | 0 |
| Part of Italy |  |  |  |
| North | Older | 24 | 50 |
|  | Younger | 24 | 50 |
| Centre | Older | 0 | 0 |
|  | Younger | 0 | 0 |
| South | Older | 0 | 0 |
|  | Younger | 0 | 0 |
| Place of Provenance |  |  |  |
| Rural area (countryside) | Older | 1 | 2 |
|  | Younger | 2 | 4 |
| Urbanized area |  |  |  |
| Metropolitan city (e.g., province) | Older | 0 | 0 |
|  | Younger | 0 | 0 |
| Medium-sized city (e.g., city) | Older | 6 | 13 |
|  | Younger | 4 | 8 |
| Small city (e.g., hamlet) | Older | 17 | 35 |
|  | Younger | 18 | 38 |
| Place of Residence |  |  |  |
| Rural area (countryside) | Older | 0 | 0 |
|  | Younger | 2 | 4 |
| Urban area |  |  |  |
| Metropolitan city (e.g., province) | Older | 0 | 0 |
|  | Younger | 1 | 2 |
| Medium-sized city (e.g., city) | Older | 4 | 8.5 |
|  | Younger | 4 | 8.5 |
| Small city (e.g., hamlet) | Older | 20 | 42 |
|  | Younger | 17 | 35 |
| Italian as mother tongue |  |  |  |
| yes | All | 48 | 100 |
| no | All | 0 | 0 |
| **Relationship with ecology** | **Group** | ***M*** | ***SD*** |
| Perceived level of expertise on ecology-related topics, from 1: "not at all" to 7: "very much" | Older | 4.62 | 1.35 |
|  | Younger | 3.88 | 1.08 |
| Perceived level of expertise on nature-related topics, from 1: "not at all" to 7: "very much" | Older | 4.79 | 1.10 |
|  | Younger | 4.00 | 1.06 |
| Estimated frequency of updates on ecology-related topics, from 1: "never" to 7: "very often" | Older | 4.83 | 1.17 |
|  | Younger | 3.71 | 1.20 |
| Estimated frequency of engagement in ecology-related activities, from 1: "never" to 7: "very often" | Older | 4.96 | 1.27 |
|  | Younger | 4.38 | 1.28 |
| Estimated frequency of engagement in green activities (e.g., gardening), from 1: "never" to 7: "very often" | Older | 5.29 | 1.52 |
|  | Younger | 3.62 | 1.88 |
| Estimated frequency of physical activity (e.g., jogging) in natural outdoors (e.g., park), from 1: "never" to 7: "very often" | Older | 5.38 | 1.38 |
|  | Younger | 4.67 | 1.66 |
| Perceived level of passion for nature, from 1: "not at all" to 7: "very much" | Older | 6.21 | 1.10 |
|  | Younger | 6.00 | 1.06 |

*Results.* Similarly to our main results (section 5.1), participants showed a high—even if slightly slower—level of accuracy in responses (Correct answers = 95%).

Results from the statistical model on RTs partially replicated previous findings. Indeed, we found a main effect of Category of Word, $\chi^{2}$(2) = 30.3235, *p* < .0001, and not a main effect of Setting, $\chi^{2}$(2) = 5.5951, *p* = .061, nor a significant interaction between Group and Setting, $\chi^{2}$(2) = 0.4721, *p* = .790. Unlike previous findings, we did not find a main effect of Group, $\chi^{2}$(1) = 1.2782, *p* = .258.

In line with main results, participants processed ecological concepts slower than both abstract, *z* = 3.427, d = 0.469, *p* = .002, and concrete concepts, *z* = 6.568, d = 0.902, *p* < .0001. Interestingly, here we found that participants processed concrete concepts faster than abstract ones, *z* = 3.121, d = 0.432, *p* = .005.

*Discussion of results.* Results replicated the main ones on the processing disadvantage of ecological upon abstract and concrete concepts (section 5.1), thus confirming that this evidence might be likely due to ecological concepts’ intrinsic nature rather than to the kind of stimuli we used as filler words (animals). Interestingly, in this case, we obtained results in line with the Concreteness Effect (Paivio, 1990), finding that concrete concepts were processed significantly faster than abstract ones for both age groups. Differences between the two tasks in the presence of such an effect might be explained by the fact that astrological entities represent a better distinct conceptual category from concrete concepts compared to animal ones (section 5.1). Indeed, animals are usually conceived as concrete concepts (Keil, 1989) and people can potentially directly experience them, as is typically true for our (concrete) artifacts. Thus, their presence might have made RTs to concrete concepts slower and obscured the Concreteness Effect, differently from what not happened with astrological concepts (section 6).

Additionally, we cannot exclude the possibility that other not-controlled variables—such as specificity/vagueness (Bolognesi et al., 2020), age of acquisition (Ellis et al., 1998), and word difficulty (see Fini et al., 2025)—may have influenced results.

Integrating these findings, the faster processing of concrete over abstract and ecological concepts seems to fit with results we gained from the feature generation task, where we found that participants, independently from age, generated more features for concrete than abstract and ecological concepts (section 5.3.1). This evidence is in line with previous research showing that the semantic richness of a domain (i.e., number of information related to a concept) might influence the elaboration speed in semantic categorization tasks. Specifically, the richness of a concept's semantic representation correlates with the formation of more robust attractors within semantic space. This phenomenon seems to facilitate a faster settling of neural activation patterns, consequently leading to an increase in response speed (Pexman et al., 2007).

Finally, as in our main results, we did not find differences in the processing speed of different kinds of concepts across different age groups and/or experimental settings, but, differently from our main results, in this case, older and younger people were not different in word processing.

***Table S1.*** *Socio-demographic information of participants.*

| **Baseline characteristics** | **Group** | **Descriptives** | |
| --- | --- | --- | --- |
|  |  | **n** | **%** |
| Sex |  |  |  |
| Male | Older | 18 | 19 |
|  | Younger | 28 | 29 |
| Female | Older | 30 | 31 |
|  | Younger | 20 | 21 |
| Intersex | Older | 0 | 0 |
|  | Younger | 0 | 0 |
| Gender |  |  |  |
| Men | Older | 16 | 17 |
|  | Younger | 26 | 27 |
| Woman | Older | 32 | 33 |
|  | Younger | 21 | 22 |
| Genderqueer | Older | 0 | 0 |
|  | Younger | 1 | 1 |
| Non-binary | Older | 0 | 0 |
|  | Younger | 0 | 0 |
| Other | Older | 0 | 0 |
|  | Younger | 0 | 0 |
| Title of study |  |  |  |
| Elementary school diploma | Older | 9 | 9 |
|  | Younger | 0 | 0 |
| Middle school diploma | Older | 11 | 12 |
|  | Younger | 0 | 0 |
| High school diploma | Older | 24 | 25 |
|  | Younger | 26 | 27 |
| Bachelor and/or Master degree | Older | 4 | 4 |
|  | Younger | 20 | 21 |
| Post-lauream title (master, PhD, etc.) | Older | 0 | 0 |
|  | Younger | 2 | 2 |
| Profession |  |  |  |
| Employed | Older | 3 | 3 |
|  | Younger | 22 | 23 |
| Student | Older | 0 | 0 |
|  | Younger | 25 | 26 |
| Unemployed | Older | 2 | 2 |
|  | Younger | 1 | 1 |
| Retired | Older | 43 | 45 |
|  | Younger | 0 | 0 |
| Socio-economic status |  |  |  |
| Extremely low income | Older | 0 | 0 |
|  | Younger | 1 | 1 |
| Low income | Older | 6 | 6 |
|  | Younger | 7 | 7.5 |
| Middle income | Older | 37 | 39 |
|  | Younger | 33 | 34 |
| High income | Older | 5 | 5 |
|  | Younger | 7 | 7.5 |
| Extremely high income | Older | 0 | 0 |
|  | Younger | 0 | 0 |
| Part of Italy |  |  |  |
| North | Older | 1 | 1 |
|  | Younger | 0 | 0 |
| Centre | Older | 47 | 49 |
|  | Younger | 48 | 50 |
| South | Older | 0 | 0 |
|  | Younger | 0 | 0 |
| Place of Provenance |  |  |  |
| Rural area (countryside) | Older | 18 | 19 |
|  | Younger | 12 | 13 |
| Urbanized area |  |  |  |
| Metropolitan city (e.g., province) | Older | 11 | 11 |
|  | Younger | 22 | 23 |
| Medium sized city (e.g., city) | Older | 4 | 4 |
|  | Younger | 4 | 4 |
| Small city (e.g., hamlet) | Older | 15 | 16 |
|  | Younger | 10 | 10 |
| Place of Residence |  |  |  |
| Rural area (countryside) | Older | 27 | 28 |
|  | Younger | 14 | 15 |
| Urban area |  |  |  |
| Metropolitan city (e.g., province) | Older | 11 | 12 |
|  | Younger | 26 | 27 |
| Medium sized city (e.g., city) | Older | 5 | 5 |
|  | Younger | 4 | 4 |
| Small city (e.g., hamlet) | Older | 5 | 5 |
|  | Younger | 4 | 4 |
| Italian as mother tongue |  |  |  |
| Yes | All | 96 | 100 |
| No | All | 0 | 0 |
| **Relationship with ecology and nature** | **Group** | ***M*** | ***SD*** |
| Perceived level of expertise on ecology-related topics,  from 1: "not at all" to 7: "very much" | Older | 4.35 | 1.30 |
|  | Younger | 4.06 | 1.28 |
| Perceived level of expertise on nature-related topics,  from 1: "not at all" to 7: "very much" | Older | 4.94 | 1.33 |
|  | Younger | 4.15 | 1.43 |
| Estimated frequency of update on ecology-related topics,  from 1: "never" to 7: "very often" | Older | 4.69 | 1.61 |
|  | Younger | 3.79 | 1.65 |
| Estimated frequency of engagement in ecology-related activities, from 1: "never" to 7: "very often" | Older | 4.94 | 1.34 |
|  | Younger | 3.77 | 1.40 |
| Estimated frequency of engagement in green activities (e.g., gardening), from 1: "never" to 7: "very often" | Older | 5.58 | 1.85 |
|  | Younger | 3.42 | 2.00 |
| Estimated frequency of physical activity (e.g., jogging) in natural outdoors (e.g., park), from 1: "never" to 7: "very often" | Older | 4.98 | 1.68 |
|  | Younger | 4.65 | 1.96 |
| Perceived level of passion for nature,  from 1: "not at all" to 7: "very much" | Older | 6.29 | 1.24 |
|  | Younger | 5.52 | 1.68 |

***Appendix B.*** *Might attitudes toward ecology and nature have affected performances of older and younger adults in the three tasks?*

In our experiment, we collected some information on the attitudes of participants toward ecology and nature (section 3.2.3), and we found that our samples of older and younger adults *a-priori* differed in some of them (section 5.4). Here, we explored whether these differences might have influenced performances the two age groups showed for ecological concepts in the categorization, rating, and feature generation tasks. Below, we detail analyses we performed (section B.1) along with results they yielded (section B.2) and a brief discussion of them (section B.3).

**B.1. Data Analysis**

To explore whether ecology and nature-related attitudes of older and younger participants might have impacted their performances for ecological concepts in the three tasks, we followed a similar statistical approach for all three kinds of behavioral data (RTs, ratings, and number of listed features). We first assessed the relationship between each attitude and the behavioral data of interest by calculating Spearman correlations (“psych” R’s package). Since the numerosity of variables to compare, we focused only on statically significant correlations equal or over |.20| (weak relationship - Dancey & Reidy, 2004). Next, for each correlation satisfying the criteria, we fitted a statistical model similar to the main model (section 4) but here featuring the behavioral data of interest related only to ecological concepts as a dependent variable, eliminating the Category of Word as a fixed factor, and inserting the ecology or nature related attitude of interest as a covariate. Specifically, for RTs, we performed generalized linear mixed models with a log-gamma distribution (“lme4” R’s package); for ratings, we fitted ordinal regression mixed models (“ordinal” R’s package); for the number of listed features, we executed generalized linear mixed models with a Poisson distribution (“lme4” R’s package).

The statistical significance of interactions between the fixed factor and the covariate for all models was determined using Type III ANOVAs (“car” R’s package for generalized linear mixed models; “RVAideMemoire” R’s package for cumulative link mixed models), and *p*-values were calculated using Wald’s Chi-squared tests. For all statistical models, comparisons across conditions were performed with Tukey’s adjustments (“emmeans” R's package).

In the case of a significant interaction between the fixed factor and the covariate, we compared each of these models (“enriched model”) to a similar model without the covariate (“main model”) through an ANOVA, to assess whether the attitude added explanatory power to data.

**B.2. Results**

*Relationship between ecology and nature-related attitudes and RTs.* We found a significant, negative, and weak Spearman correlation only between RTs and the estimated frequency of engagement in *green* activities, *r_s_*(3723) = -.31, *p* < .0001. Despite this, the model enriched with this covariate showed no significant interactions between this attitude with Group, $\chi^{2}$(1) = 1.1862, *p* = .276, with Setting, $\chi^{2}$(2) = 0.5091, *p* = .775, with Group and Setting, $\chi^{2}$(2) = 1.9563, *p* = .376.

*Relationship between ecology and nature-related attitudes and ratings.* We found that only Word Confidence, Familiarity, and Perceived Distance dimensions had a significant relationship with ecology and nature-related attitudes. Specifically, we found significant, positive, and weak correlations between Word Confidence and the perceived level of expertise on ecology, *r_s_*(958) = .37, *p* < .0001, the perceived level of expertise on nature, *r_s_*(958) = .29, *p* < .0001, the estimated frequency of updates on ecological topics, *r_s_*(958) = .27, *p* < .0001, the estimated frequency of engagement in ecology-related activities, *r_s_*(958) = .30, *p* < .0001, and the estimated frequency of engagement in outdoor activities, *r_s_*(958) = .23, *p* < .0001. We also found significant, positive, and weak correlations between Familiarity and the perceived level of expertise on ecology, *r_s_*(958) = .22, *p* < .0001, and the estimated frequency of engagement in ecology-related activities, *r_s_*(958) = .20, *p* < .0001. Finally, we found significant, negative, and weak correlations between Perceived Distance and the perceived level of expertise on ecology, *r_s_*(958) = .23, *p* < .0001, the perceived level of expertise on nature, *r_s_*(958) = .25, *p* < .0001, the estimated frequency of engagement in ecology-related activities, *r_s_*(958) = .24, *p* < .0001, the estimated frequency of engagement in *green* activities, *r_s_*(958) = .25, *p* < .0001, and the perceived level of passion towards nature, *r_s_*(958) = .21, *p* < .0001.

Despite this, the models enriched with the covariates showed no significant interaction between these characteristics and Group (Table B.1), apart from the two models targeting Word Confidence and Perceived Distance ratings as a dependent variable and the perceived level of expertise on ecology as a covariate, that showed a significant interaction between these attitudes and Group, Model on Word Confidence: $\chi^{2}$(1) = 4.7126, *p* = .030; Model on Perceived Distance: $\chi^{2}$(1) = 5.2352, *p* = .022.

As for the model on Word Confidence, simple slope analysis revealed that both the slopes of older (LCL 0.764 – UCL 1.533, estimate = 1.148) and younger adults (LCL 0.181 – UCL 0.928, estimate = 0.555) were significantly different from zero as a function of the factor ecological expertise, and they significantly differ between each other, *z* = 2.189, SE = .271, *p* = .029 (Figure B.1, Panel A).

As for the model on Perceived Distance, simple slope analysis revealed that only the slope of older adults was significantly different from zero as a function of the factor ecological expertise (LCL -1.17 – UCL -0.388, estimate = -0.778; younger adults’ slope: LCL -0.52 – UCL 0.236, estimate = -0.142), and significantly differed from that of younger adults, *z* = -2.300, SE = .277, *p* = .022 (Figure B.1, Panel B).

Despite these significant results, only the comparison between the enriched model on Word Confidence and the relative main model showed that the former added significantly more explanatory power to the latter, $\chi^{2}$(2) = 36.717, *p* < .0001 (Enriched Model on Perceived Distance *vs* Main Model on Perceived Distance: $\chi^{2}$(2) = -255.75, *p* = 1.000).

*Table B.1. Anova results of the interaction between each ecology or nature-related attitude and the experimental group in each statistical model performed on dimensions that were significantly correlated with the attitude of interest. Significant interactions are marked in bold. The sign “//” indicates that we did not perform the statistical model comparing the attitude and the dimension of interest since the two variables did not satisfy our correlation criteria.*

| **Ecology or nature-related attitude** | **Model on Word Confidence** | **Model on Familiarity** | **Model on Perceived Distance** |
| --- | --- | --- | --- |
|  | **Interaction with Group:** | | |
| Perceived level of expertise in ecology | $\chi^{2}$**(1) = 4.7126, *p* = .030** | // | $\chi^{2}$**(1) = 5.2352, *p* = .022** |
| Perceived level of expertise on nature | $\chi^{2}$(1) = 0.117953, *p* = .731 | $\chi^{2}$(1) = 1.29264, *p* = .256 | $\chi^{2}$(1) = 0.72874, *p* = .393 |
| Estimated frequency of updates on ecology-related topics | $\chi^{2}$(1) = 0.37271, *p* = .542 | // | // |
| Estimated frequency of engagement in ecology-related activities | $\chi^{2}$(1) = 0.63765, *p* = .425 | $\chi^{2}$(1) = 1.6869, *p* = .194 | $\chi^{2}$(1) = 1.02182, *p* = .312 |
| Estimated frequency of engagement in green activities | // | // | $\chi^{2}$(1) = 0.0095, *p* = .922 |
| Estimated frequency of outdoor activities | $\chi^{2}$(1) = 0.48541, *p* = .486 | // | // |
| Perceived level of passion for nature | // | // | $\chi^{2}$(1) = 0.26131, *p* = .609 |


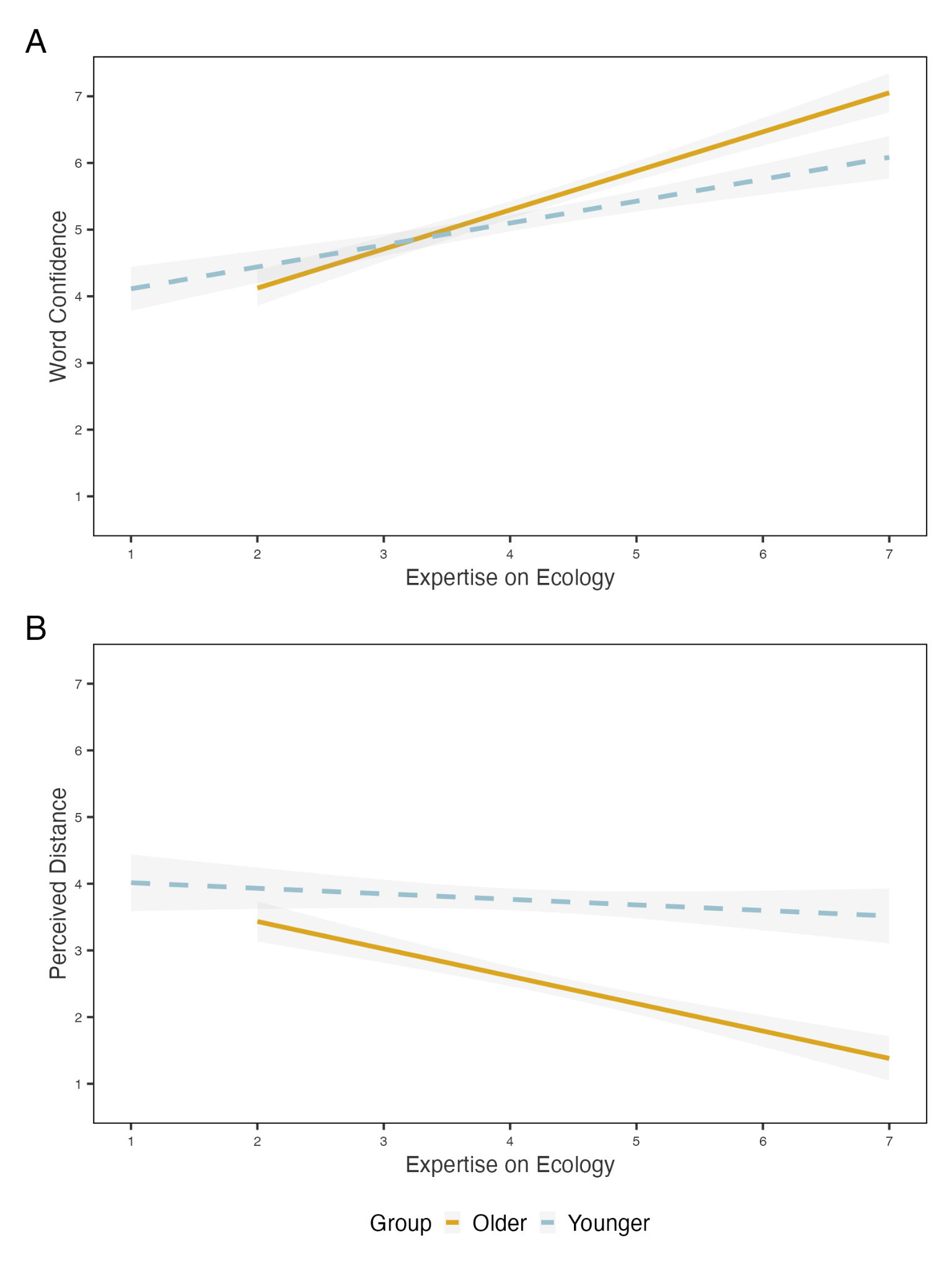


**Figure B.1.** Ratings on Word Confidence (**Panel A**) and Psychological Distance (**Panel B**) to ecological concepts which older and younger adults showed at increased levels of expertise on ecological topics.

*Relationship between ecology and nature-related attitudes and the number of listed features.* We found significant, positive, and weak Spearman correlations between the number of listed features and the estimated frequency of engagement in outdoor activities, *r_s_*(94) = .26, *p* = .012, and the perceived level of passion towards nature, *r_s_*(94) = .24, *p* = .019.

Despite this, the models enriched with these two attitudes as covariates showed no significant interactions between these characteristics and Group, Estimated frequency of engagement in outdoor activities, $\chi^{2}$(6) = 1.7194, p = .944; Perceived level of passion towards nature, $\chi^{2}$(5) = 2.7742, p = .735.

**B.3. Discussion of results**

Despite the relevant differences we found in ecology and nature related attitudes in older and younger adults (section 5.4), here we found that these attitudes seem to have slightly modulated performances of participants in the three tasks, with a higher impact on the rating than categorization and feature generation tasks.

To illustrate, for the categorization task, we only found a negative but weak relationship between RTs and frequency of engagement in *green* activities, suggesting that the more participants perceived to be frequently committed to *green* activities, such as gardening, the faster the processing speed of ecological concepts was. Similarly, in the feature generation task, we found two positive but weak correlations between the number of listed features and the estimated frequency of engagement in outdoor activities and the perceived level of passion towards nature, suggesting that the more participants felt to be frequently engaged in outdoor activities in nature, such as jogging, or the more they loved nature, the higher the number of features they listed for ecological concepts.

Thus, our data revealed that only nature-related attitudes correlated with performances for ecological concepts in the two tasks. These results might be explained by the fact that since ecology and nature are strictly intertwined topics, being highly devoted to nature (e.g., spending considerable time in nature, frequently engaging in *green* activities or, more broadly, being highly passionate to natural beings) might bring more knowledge and familiarity with climate change themes, which, in turn, might make ecological concepts easier to be processed or denser in their semantic representation. However, for both tasks, we did not find any direct effect of these attitudes on the performances shown by older and younger adults.

Interestingly, we found more numerous relationships between ecology and nature-related attitudes and rating scores, specifically those concerning Word Confidence, Familiarity, and Perceived Distance dimensions. To illustrate, we first found that the more participants perceived to be experts in ecology or nature, frequently updated on these topics, and frequently involved in ecological actions and outdoor activities in nature, the more they perceived to master ecological concepts meaning. Similarly, the more participants perceived to be experts in ecology and frequently involved in ecological activities, the more they considered ecological concepts familiar. Finally, the more participants perceived to be experts in ecology or nature, frequently involved in ecological and green activities, and passionate about nature, the more they perceived ecological concepts closely. In other words, the more participants had higher levels of interest, experience and expertise with the domain, the more ecological concepts were familiar, well-mastered and psychologically close.

Within these attitudes, only expertise on ecology showed to differently modulate ratings of older and younger individuals on Word Confidence and Perceived Distance.

In the case of Word Confidence, we found that expertise significantly impacted ratings of both age groups, but with a higher effect on older people. Indeed, at increased perceived levels of expertise in ecology, both age groups felt more confident in mastering ecological concepts’ meaning, but older adults significantly more than younger adults (Figure B.1, Panel A). Interestingly, our results also showed that the model with ecological expertise as a covariate explained data better than the main model without this attitude as a covariate, thus suggesting that expertise might be an important factor mediating the relationship between individuals and their level of confidence in mastering the meaning of a new and not still well-defined domain such as that of ecology, especially if they are older adults.

In the case of Perceived Distance, we found that expertise impacted the ratings of only older adults. Indeed, at increased levels of expertise on ecology, older adults perceived ecological concepts as increasingly psychologically closer. Conversely, for younger adults, increasing levels of expertise did not convert into an increased psychological closeness to ecological concepts (Figure B.1, Panel B). This data might contribute to integrate previous evidence showing a positive link between psychological closeness and willingness to act (Leviston et al., 2014), making us hypothesize that the relationship between psychological closeness to ecological themes and motivation to positively behave toward the environment might be influenced by the level of expertise that someone perceives on ecology. However, this complex relationship might depend on people’s age. Indeed, at increasing levels of expertise in ecology, younger adults might continue to feel ecological phenomena far from them which, in turn, might not convert into actual pro-environmental behaviors. Conversely, expertise might be crucial for older adults. Indeed, it might help older adults perceive ecological phenomena closer and thus better motivate them to act eco-friendly. Hence, these results might concur to explain why younger adults, despite being, on average, more interested—that is, more informed, thus, more expert—in ecological topics, are also less willing to engage in actual pro-environmental behaviors than older adults (Wiernik et al., 2013).

***Table S2.*** *Categories of concepts, Italian words, and their English equivalents. These represent the linguistic stimuli used in the preregistered and “control” (Appendix A, SM) categorization tasks.*

| **Category of concepts** | **Italian Words** | **English Translations** |
| --- | --- | --- |
| **Stimuli of the Learning Phase** | | |
| **Abstract** | *Antichità; Categoria; Destino; Enigma; Filosofia.* | *Ancient Times; Category; Fate; Enigma; Philosophy.* |
| **Concrete** | *Arma; Cera; Denaro; Falce; Scopa.* | *Weapon; Wax; Money; Sickle; Broom.* |
| **Ecological** | *Danno Ambientale; Desertificazione; Polveri Sottili; Riserva Naturale; Surriscaldamento.* | *Environmental Damage; Desertification; Fine Dust; Nature Reserve; Overheating.* |
| **Animal (preregistered task)** | *Canarino; Carpa; Pinguino; Talpa; Zebra.* | *Canary; Carp; Penguin; Mole; Zebra.* |
| **Astrology (“control” task)** | *Ammasso Stellare; Astrolabio; Legge Di Gravità; Navicella Spaziale; Corpo Nero.* | *Star Cluster; Astrolabe; Law Of Gravity; Spacecraft; Black Body.* |
| **Stimuli of the Main Experiment** | | |
| **Abstract** | *Affermazione; Amarezza; Analogia; Assoluzione; Attitudine; Avarizia; Circostanza; Concordanza; Consapevolezza; Credenza; Deduzione; Definizione; Democrazia; Denigrazione; Derivazione; Descrizione; Dimenticanza; Disonestà; Elezione; Esitazione; Fantasma; Fervore; Fremito; Impunità; Indolenza; Infinito; Insolenza; Mistero; Nazione; Paradiso; Poetica; Pretesto; Purezza; Religione;*  *Salvezza; Scrupolo; Tirannide; Tregua; Unanimità; Vicenda.* | *Affirmation; Bitterness; Analogy;*  *Absolution; Attitude; Greed;*  *Circumstance; Concordance; Awareness;*  *Belief; Deduction; Definition;*  *Democracy; Disparagement; Derivation; Description; Oblivion; Dishonesty;*  *Election; Hesitation; Ghost; Fervour;*  *Thrill; Impunity; Indolence; Infinity;*  *Insolence; Mystery; State; Paradise;*  *Poetics; Pretence; Purity; Religion;*  *Salvation; Scruple; Tyranny; Truce;*  *Unanimity; Story.* |
| **Concrete** | *Argento; Automobile; Bandiera; Bicchiere; Bicicletta; Bottiglia; Calzino; Cappello; Cemento; Coltello; Cravatta; Cristallo; Cucchiaio; Diamante; Elicottero; Fontana; Forbici; Forchetta; Libreria; Martello; Matita; Ombrello; Orologio; Pantaloni; Pennello; Poltrona; Polvere; Romanzo; Scatola; Scrivania; Specchio; Statua; Stivale; Sveglia; Tavolo; Telegrafo; Trapano;*  *Trattore; Uniforme; Università.* | *Silver; Car; Flag; Glass;*  *Bicycle; Bottle; Sock; Hat;*  *Cement; Knife; Tie; Crystal;*  *Spoon; Diamond; Helicopter; Fountain;*  *Scissors; Fork; Bookcase; Hammer;*  *Pencil; Umbrella; Clock; Trousers;*  *Brush; Armchair; Dust; Novel;*  *Box; Desk; Mirror; Statue; Boot;*  *Alarm Clock; Table; Telegraph; Drill;*  *Tractor; Uniform; University.* |
| **Ecological** | *Agricoltura; Allevamento; Ambiente; Biodiversità; Buco Dell' Ozono;*  *Cibo Biologico; Clima; Combustibile; Compost; Concime; Deforestazione; Depuratore; Discarica; Ecosistema;*  *Effetto Serra; Emissioni; Energia; Fauna; Flora; Fognatura; Fumi; Gas; Gas Serra; Ghiacciaio; Impatto Zero; Industria; Inquinamento; Metalli Pesanti; Pale Eoliche; Pesticidi; Petrolio; Piogge Acide; Plastica; Radiazioni; Riciclaggio; Rifiuti; Riuso; Scorie; Smog; Temperatura.* | *Agriculture; Livestock; Environment; Biodiversity; Ozone Hole;*  *Organic Food; Climate; Combustible;*  *Compost; Fertiliser; Deforestation;*  *Purifier; Rubbish Dump; Ecosystem;*  *Greenhouse Effect; Emissions; Energy; Fauna; Flora; Sewer; Fumes; Gas; Greenhouse Gases; Glacier; Zero Impact; Industry;*  *Pollution; Heavy Metals; Wind Turbines; Pesticides; Petroleum; Acid Rain; Plastic; Radiations; Recycling; Waste; Reuse;*  *Dross; Smog; Temperature.* |
| **Animal (preregistered task)** | *Aquila; Balena; Cammello; Cane; Capra; Cavallo; Cervo; Cicogna; Cinghiale; Civetta; Coccodrillo; Coniglio; Delfino; Elefante; Falco; Farfalla; Foca; Gabbiano; Gallina; Gallo; Gatto; Gufo; Leone; Leopardo;*  *Lupo; Maiale; Mucca; Oca;*  *Orso; Pappagallo; Pecora; Piccione; Rondine; Scimmia; Serpente; Squalo; Tigre; Topo; Toro; Volpe.* | *Eagle; Whale; Camel; Dog; Goat;*  *Horse; Deer; Stork; Boar;*  *Owl; Crocodile; Rabbit; Dolphin;*  *Elephant; Hawk; Butterfly; Seal;*  *Seagull; Chicken; Cock; Cat; Owl; Lion;*  *Leopard; Wolf; Pig; Cow; Goose;*  *Bear; Parrot; Sheep; Pigeon;*  *Swallow; Monkey; Snake; Shark; Tiger;*  *Mouse; Bull; Fox.* |
| **Astrology (“control” task)** | *Asteroide; Astro; Astrofisica; Astrologia; Astronauta; Astronave; Big Bang;*  *Buco Nero; Cometa; Corona Solare; Plenilunio; Cosmo; Cosmologia;*  *Costellazione; Cratere Lunare; Eclissi; Galassia; Luce Solare; Luna; Macchie Solari; Materia Oscura; Meteora; Meteorite; Nana Bianca; Stazione Spaziale; Nebulosa;*  *Orbita; Pianeta; Protostella; Satellite; Sfera Celeste; Sistema Solare; Sole; Solstizio; Stella; Supernova; Universo; Via Lattea; Zodiaco.* | *Light Year; Asteroid; Star; Astrophysics; Astrology; Astronaut; Spaceship; Big Bang; Black Hole; Comet; Solar Corona;*  *Full Moon; Cosmos; Cosmology;*  *Constellation; Luna Crater; Eclipse;*  *Galaxy; Sunlight; Moon; Sunspots;*  *Dark Matter; Meteor; Meteorite;*  *White Dwarf; Space Station; Nebula;*  *Orbit; Planet; Protostar; Satellite; Celestial Sphere; Solar System; Sun; Solstice;*  *Star; Supernova; Universe; Milky Way;*  *Zodiac.* |

***Table S3.*** *Categories of concepts, Italian words, and their English equivalents. These represent the linguistic stimuli used in the feature generation and rating tasks.*

| **Category of concepts** | **Italian Words** | **English Translations** |
| --- | --- | --- |
| **Abstract** | *Analogia; Credenza; Deduzione; Democrazia; Infinito; Mistero; Paradiso; Purezza;*  *Religione; Salvezza.* | *Analogy; Belief; Deduction; Democracy; Infinity; Mystery; Paradise; Purity;*  *Religion; Salvation.* |
| **Concrete** | *Calzino; Cravatta; Fontana; Matita; Ombrello; Statua; Stivale; Sveglia; Tavolo; Trattore.* | *Sock; Tie; Fountain; Pencil; Umbrella;*  *Statue; Boot; Alarm Clock; Table; Tractor.* |
| **Ecological** | *Ambiente; Biodiversità; Clima;*  *Ecosistema; Energia; Fauna; Impatto Zero;*  *Pale Eoliche; Riciclaggio; Riuso.* | *Environment; Biodiversity; Climate; Ecosystem; Energy; Fauna; Zero Impact;*  *Wind Turbines; Recycling; Reuse.* |

***Table S4.*** *Shared elements of the testing places included in the three experimental settings (Indoor, Natural Outdoor, Urbanized Outdoor).*

| **Indoor setting** | **Natural setting** | **Urbanized setting** |
| --- | --- | --- |
| Staying in the kitchen, the living room, or the laboratory  (visuo-auditory stimulation:  mainly artificial and static: chairs, table, sofa, TV, wall, carpet, quiet contexts). | Sitting outside in the house’s garden or in a park  (visuo-auditory stimulation mainly natural and dynamic: trees, flowers, plants, children playing, dogs, people walking). | Sitting on the balcony or on a bench in a city square.  (visuo-auditory stimulation mainly artificial and dynamic: car noise, people working, urban infrastructure). |

***Table S5.*** *Means (M) and Standard Deviations (SD) of Reaction Times (in ms) shown by Older and Younger participants for Abstract, Concrete, and Ecological concepts in the Indoor, Natural Outdoor and Urbanized Outdoor settings.*

| **Category of Word** | **Setting** | **Older Adults** | | **Younger Adults** | |
| --- | --- | --- | --- | --- | --- |
|  |  | ***M*** | ***SD*** | ***M*** | ***SD*** |
| Abstract | Indoor | 1009.06 *ms* | 420.24 *ms* | 711.16 *ms* | 242.58 *ms* |
|  | Natural Outdoor | 860.96 *ms* | 274.16 *ms* | 738.84 *ms* | 290.09 *ms* |
|  | Urbanized Outdoor | 845.78 *ms* | 211.15 *ms* | 804.99 *ms* | 327.40 *ms* |
| Concrete | Indoor | 978.90 *ms* | 369.09 *ms* | 699.03 *ms* | 221.41 *ms* |
|  | Natural Outdoor | 859.33 *ms* | 238.02 *ms* | 709.85 *ms* | 239.83 *ms* |
|  | Urbanized Outdoor | 844.08 *ms* | 206.90 *ms* | 805.53 *ms* | 329.42 *ms* |
| Ecological | Indoor | 1078.08 *ms* | 432.42 *ms* | 747.81 *ms* | 255.79 *ms* |
|  | Natural Outdoor | 920.14 *ms* | 292.76 *ms* | 760.76 *ms* | 272.33 *ms* |
|  | Urbanized Outdoor | 891.97 *ms* | 244.82 *ms* | 830.68 *ms* | 327.81 *ms* |

***Table S6.*** *Cronbach’s Alphas (*ɑ*) of Interrater Reliability of All the Targeted Dimensions for the two age cohorts*.

|  | **Group** | | |
| --- | --- | --- | --- |
|  | **Older** |  | **Younger** |
| **Dimension** | ***ɑ*** |  | ***ɑ*** |
| Age of Acquisition (AoA) | 0.89 |  | 0.89 |
| Concreteness~Abstractness (ABS) | 0.87 |  | 0.93 |
| Familiarity (FAM) | 0.95 |  | 0.92 |
| Openness to Negotiation (ON) | 0.95 |  | 0.95 |
| Perceived Distance (PD) | 0.91 |  | 0.92 |
| Social Metacognition (SM) | 0.94 |  | 0.95 |
| Word Confidence (WC) | 0.95 |  | 0.94 |

**Table S7.** *Similarities and differences in ratings provided by older and younger participants to Ecological concepts and to Ecological concepts in relation to Abstract and Concrete concepts on targeted dimensions. Dimensions are reported in alphabetic order. In the table, the minus sign (“-”) indicates that Ecological concepts received a statistically significant lower score than the Contrasted Category of Word; The plus sign (“+”) indicates that the Ecological concepts received a statistically significant higher score than the Contrasted Category of Word; The equal sign (“=”) indicates that the score to Ecological concepts did not statistically differ from that of the Contrasted Category of Word. Finally, the “//” sign indicates no reasonable comparison between the contrasted categories of concepts.*

| **Dimension** | **Group** | **Ecological Concepts Contrasted with:** | | |
| --- | --- | --- | --- | --- |
|  |  | **Abstract**  **Concepts** | **Concrete**  **Concepts** | **Ecological Younger Concepts** |
| Age of Acquisition  (here, level of late acquisition) | Older | + | + | + |
|  | Younger | = | + | // |
| Concreteness~Abstractness  (here, level of abstractness) | Older | - | + | = |
|  | Younger | - | + | // |
| Familiarity  (here, level of familiarity) | Older | = | - | = |
|  | Younger | = | - | // |
| Openness to Negotiation  (here, level of openness to negotiate the conceptual meaning) | Older | - | + | = |
|  | Younger | - | + | // |
| Perceived Distance  (here, level of psychological distance felt from the concept) | Older | - | + | - |
|  | Younger | - | + | // |
| Social Metacognition  (here, level of need of the others’ help to understand the concept’s meaning) | Older | = | + | = |
|  | Younger | = | + | // |
| Word Confidence  (here, level of confidence in mastering the concept’s meaning) | Older | = | - | + |
|  | Younger | = | - | // |

***Appendix C.*** *Similarities and differences between older and younger adults in the knowledge related to “Recycling” —insights from the topics encompassed by their semantic networks’ communities.*

*Older Adults’ Network.* The first aspect of interest in this network is the coexistence of a positive *vs.* negative vision of recycling. Indeed, there are two main communities positively characterizing it (light blue community: e.g., *“Right”; “Beautiful”.* Light orange community: *“Engaging”; “Respectable”*) and mainly highlighting its beneficial outcomes for both people and nature (light blue community: e.g., *“Saving”; “Pro-Nature”*. Light orange community: e.g., *“Less-Waste”, “Not-Polluting”*). These communities oppose a more numerous sets with a predominantly negative connotation of recycling, mainly due to its complexity, lack of organization, difficulty in the implementation, inutility, waste of energy and money (purple community: e.g., *“Demanding”; “Impossible”*. Dark orange community: e.g., *“Disorganized”; “Useless”; “Expensive”; “Unclear”.* Red community: e.g., *“Ineffective”; “Confusing”*) and to the illicit aspect of recycling that in Italy is usually thought to be related to mafia activities (light cherry community: e.g., *“Penal”; “Multi-Managed”*. Dark blue community: e.g., *“Of Money”; “Illicit”*).

Another salient topic in the older network focuses on the practical aspects of functioning and the processing technique of recycling, mostly related to giving new life to objects (petroleum community: e.g., *“Recomposed”; “Regenerated”*). In line with this, two other communities stress the action of reuse recycling can imply (light green community: e.g., *“Recovered”; “Transformed”*. Dark green community: e.g., *“Reused”; “Refurbished”*).

Finally, in this network, we also find two communities containing the kinds of entities that can be objects of recycling (light green community: e.g., *“Of Clothes”; “Food”*. Dark blue community: e.g., *“Alimentary”; “Electronic”*), and a final, multi-thematic, one (i.e., dark blue community) empathizing the thematic sphere of recycling (*“Ecological”; “Green”*), its social value (*“Social”; “Reciprocal”*), and its obligatoriness (*“Coercive”; “Controlled”*) (see Figure C.1, Panel A).

*Younger Adults’ Network.* As in the older network, also in the younger one, we first find an opposition between a positive *vs.* negative vision of recycling. Indeed, there are two positively connotated communities mainly highlighting recycling’s clearness and efficiency-related facets (dark green community: e.g., *“Understandable”; “Effective”*. Light red community: e.g., *“Beneficial”; “Capable”*) along with other two communities emphasizing the advantage recycling can bring to people and nature (light orange community: e.g., *“Helpful”; “Pro-Nature”*). Interestingly, differently from older adults, in relation to positive outcomes for humans, younger adults seem also to include an opposition between individual and societal benefits (personal benefits: light orange community: e.g., *“Personal”; “Individual”*. Societal benefits: light green community: e.g., *“Societal”; “Community”*. Light orange community: *“Collective”; “Common”*). All these more positively connotated communities oppose a lower number of more negatively valenced ones, which mostly stress the illicit aspect of recycling related to mafia activities (dark orange community: e.g., *“Immoral”; “Incriminating”*. Red community: *“Hidden”; “Penal”*. Light blue community: *“Of Money”; “Criminal”*).

In a similar vein, as in the older network, one community relates to the kind of objects that can be recycled (extra-dark orange community: e.g., *“Of Non-Recyclable Waste”; “Of Plastic”*) and another to the thematic sphere of recycling (light blue community: e.g., *“Environmental”; “Ecological”*). Similarly, two further communities relate to the functioning aspects of recycling—with a higher emphasis on its efficiency in younger compared to older adults (purple community: e.g., *“Improved”; “Worsened”*) —, and to its obligatoriness—with younger adults also emphasizing the voluntary aspect of recycling compared to older adults (light blue community: e.g., *“Mandatory”; “Voluntary”*). Finally, two last communities include topics that are exclusive to younger participants: the importance of recycling along with the efforts it requires (brown community: *“Fundamental”; “Tiring”*) and its ampleness/diffusion (red community: *“Vast”; “Widespread”*) (see Figure C.1, Panel B).


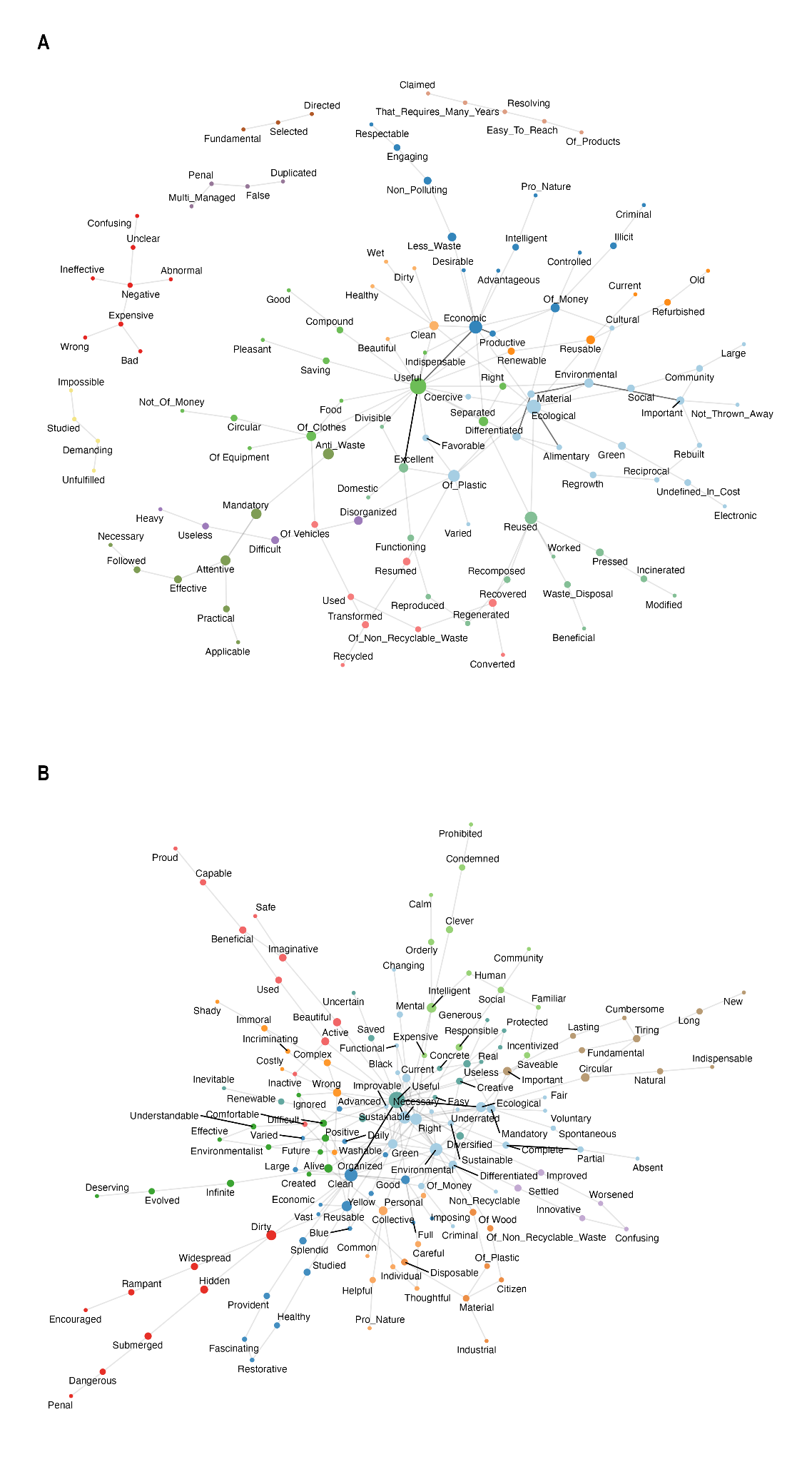
**Figure C.1.** Network of features listed by older (**Panel A**) and younger adults (**Panel B**) for “Recycling”. In the networks, the size of nodes (features) indicates their centrality, their color identifies the community to which they belong, and the thickness of the links indicates the frequency of co-occurrence of bigrams.

**References of the Supplementary Materials**

Bolognesi, M., Burgers, C., & Caselli, T. (2020). On abstraction: decoupling conceptual concreteness and categorical specificity. Cognitive Processing, *21*(3), 365-381.

Dancey, C. and Reidy, J. (2004) *Statistics without Maths for Psychology: using SPSS for Windows*. Prentice Hall, London.

Ellis, A. W., & Morrison, C. M. (1998). Real age-of-acquisition effects in lexical retrieval. Journal of experimental psychology: learning, Memory, and cognition, *24*(2), 515.

Keil, F. C. (1989). *Concepts, kinds, and cognitive development*. The MIT Press.

Pexman, P. M., Hargreaves, I. S., Edwards, J. D., Henry, L. C., & Goodyear, B. G. (2007). The neural consequences of semantic richness. *Psychological science*, *18*(5), 401-406.
